# Torsemide Pharmacometrics in Healthy Adult Populations Including CYP2C9 Genetic Polymorphisms and Various Patient Groups through Physiologically Based Pharmacokinetic-Pharmacodynamic Modeling

**DOI:** 10.3390/pharmaceutics14122720

**Published:** 2022-12-05

**Authors:** Seung-Hyun Jeong, Ji-Hun Jang, Yong-Bok Lee

**Affiliations:** 1 College of Pharmacy, Sunchon National University, 255 Jungang-ro, Suncheon-si 57922, Jeollanam-do, Republic of Korea; 2 College of Pharmacy, Chonnam National University, 77 Yongbong-ro, Buk-gu, Gwangju 61186, Republic of Korea

**Keywords:** torsemide, pharmacokinetic (PK), pharmacodynamic (PD), physiologically based pharmacokinetic-pharmacodynamic (PBPK-PD) modeling, CYP2C9

## Abstract

Torsemide is a widely used diuretic in clinical practice. In this study, pharmacokinetic (PK) and pharmacodynamic (PD) simulations of torsemide for various population groups and exposure scenarios were performed through human-scale physiologically-based PK-PD (PBPK-PD) modeling of torsemide. For PBPK-PD modeling of torsemide, invitro and clinical data of torsemide reported previously were used. After exposure to clinical doses of torsemide, observed plasma (or serum) concentration and urine torsemide excretion profiles were used as PK-data, and observed urinary sodium excretion rate was used as PD-data. The model was then extended to take into account physiological and biochemical factors according to different CYP2C9 phenotypes or patient populations. The established model captured various torsemide clinical results well. Differences in torsemide PKs and PDs between patient groups or CYP2C9 genetic polymorphisms were modelologically identified. It was confirmed that degrees of differences in torsemide PKs and PDs by disease groups were greater than those according to different CYP2C9 phenotypes. According to torsemide administration frequency or dose change, it was confirmed that although the difference in plasma PKs between groups (healthy adult and patient groups) could increase to 14.80 times, the difference in PDs was reduced to 1.01 times. Results of this study suggested that it is very important to consider disease groups in the setting of torsemide clinical therapy and that it is difficult to predict PD proportionally with only differences in PKs of torsemide between population groups. The PBPK-PD model established in this study is expected to be utilized for various clinical cases involving torsemide application in the future, enabling optimal drug therapy.

## 1. Introduction

Torsemide is a diuretic used clinically to treat fluid overload caused by congestive heart failure (CHF), chronic kidney disease (CKD), liver disease (cirrhosis), and high blood pressure [1]. It is generally classified as a class I drug with high solubility and high permeability according to the biopharmaceutics classification system (BCS) [2]. Torsemide has a diuretic effect by blocking the Na^+^/K^+^/2Cl^−^ carrier system in the luminal cell membrane of the ascending loop of Henle [3]. According to previous reports [4,5,6], the metabolism of torsemide in the body occurs mainly in the liver, and its action mechanism is known to be related to the oxidation reaction by CYP2C9 (Cytochrome P450 family 2 subfamily C member 9). Therefore, the degree of torsemide metabolism differs depending on genetic polymorphisms of CYP2C9, a major factor influencing the pharmacokinetic (PK) variability between individuals within a population [5,6,7]. The major in vivo metabolite of torsemide has been reported to be pharmacologically inactive [1]. Therefore, it has been judged that the metabolite of torsemide is not significantly related to its pharmacological effect, that is, urinary sodium excretion.

When torsemide is administered orally, the time to reach the maximum blood concentration through the gastrointestinal (GI)-tract is fast (within 2–3 h) [1,7]. In healthy adult populations, the half-life of torsemide is known to be very fast (3–6 h) [1,7]. Plasma protein binding of torsemide has been reported to be 90% or higher [1]. In the past in vitro test [8] for human serum albumin (HSA), it was confirmed that torsemide protein binding was 95% on average (at torsemide concentration of 1 to 50 μg/mL). Although in vitro results were obtained from rat blood [8], it confirmed that the distribution of torsemide from plasma to blood cells was 0.279 on average (at torsemide concentration of 1 to 10 μg/mL). It has been reported that PKs of torsemide is linear at doses up to 200 mg in healthy adult or patient populations [1]. The dose-independency of torsemide PKs has also been confirmed in in vivo experiments using rats [8]. Regarding information about the pharmacodynamics (PDs) of torsemide, the degree of sodium excretion through urine has been mainly reported [9,10,11,12,13,14,15]. This might be because the pharmacological mechanism of torsemide is closely related to the fact that torsemide exerts a diuretic effect through sodium excretion from the body [1]. The site of action of torsemide (as Na^+^/K^+^/2Cl^−^ carrier) is directly related to its excretion route through urine, which might be an important reason [14].

The main purpose of this study was to perform physiologically based pharmacokinetic-pharmacodynamic (PBPK-PD) modeling based on in vitro [4,6,8] and clinical data of torsemide reported previously [7,9,10,11,16,17,18,19,20,21]. PBPK-PD modeling for torsemide enables the simulation of multiple scenarios. It can serve as a basis for the effective clinical therapy setting of torsemide. Although many clinical studies on torsemide have been conducted [9,10,11,16,17,18,19,20,21], studies on its dosage and usage prediction based on modeling are very scarce. Quantitative associations between torsemide PKs and PDs were not readily identified. As a result, the prediction of torsemide PKs and PDs for different patient groups or individuals was difficult. Therefore, torsemide PK and PD variabilities in healthy adult and patient populations need to be described as a linked model to establish a scientific basis for a case-specific clinical therapy setting. PBPK-PD modeling has the advantage of being able to predict drug PKs and PDs similarly to actual body systems by integrating biological and physiological information at the organism level [22]. One of the great advantages of PBPK-PD modeling is that it can be simulated relatively easily for various scenarios (such as different administration protocols and modified physiological systems). Therefore, torsemide clinical therapy studies in various population groups were attempted, utilizing the advantages of PBPK-PD modeling.

The ultimate goal of pharmacotherapy is to identify factors of variability in drug response and appropriately control it to achieve maximum effectiveness. This is also the ultimate goal of individual pharmacotherapy or precision medicine [23]. Through this study, key considerations in torsemide clinical application were identified. PKs and PDs prediction (of torsemide) could be effectively used for virtual populations when it is difficult to conduct actual clinical trials. The torsemide PBPK-PD model established in this study can be utilized to establish the optimal torsemide dose and regimen for various adult populations. Finally, the modeling approach in this study provides an important hint toward the establishment of torsemide’s personalized pharmacotherapy and/or precision medicine.

## 2. Materials and Methods

### 2.1. Data Collection

To establish a human-scale PBPK-PD model for torsemide, data from previously reported clinical studies [5,7,9,10,11,12,13,15,16,17,18,19,20,21,24,25,26] were extensively collected. PK and PD results of torsemide performed on healthy adult populations (with consideration of different CYP2C9 phenotypes), CKD, cirrhosis, and CHF patients were also collected. Most data came from studies on male adults. Some were mixed with data from female adults. Therefore, all data regardless of gender were integrated and used for modeling. When the identification of data information was uncertain or the presentation of interpretable units was insufficient, results were excluded from this study. To establish a reliable torsemide PBPK-PD model, it is very important to acquire PK and PD profile patterns according to the torsemide exposure time. Most results of torsemide plasma concentration, urine excretion rate, and urine sodium excretion rate presented graphically in the literature were mean or median values of the group. These were quantified through data reading. Urinary torsemide excretion rate data could be converted to the excreted amount by multiplying the observed time. All graph results were digitized using WebPlotDigitizer (version 4.5). In addition, results of individual torsemide bioequivalence tests performed on multiple healthy adults in the past were used together for modeling in this study. Finally, all collected data were integrated into group-specific data sets and applied for modeling. Table S1 presents basic information on the data used for torsemide PBPK-PD modeling.

### 2.2. Workflow of Torsemide PBPK-PD Modeling

**Figure 1:**
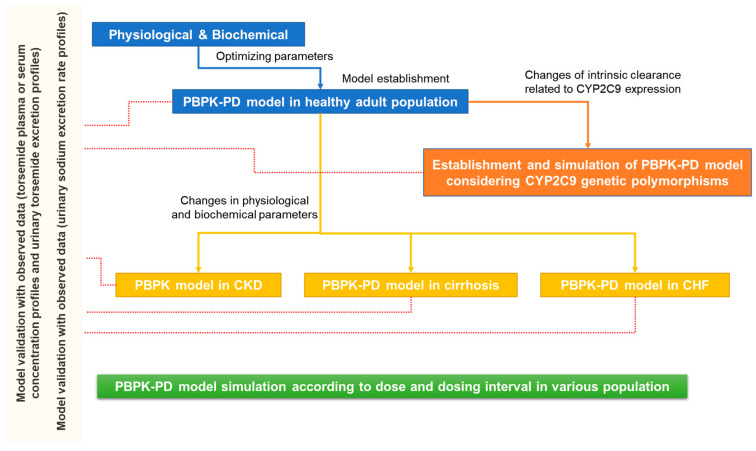
Workflow of torsemide PBPK-PD modeling.

In this study, a PBPK model was established primarily based on torsemide PK results (plasma or serum torsemide concentration and urinary excretion according to torsemide exposure) in healthy adult populations. Torsemide PD results (rates of urinary sodium excretion following torsemide exposure; baseline sigmoid E_max_ model) were linked to the torsemide PBPK model through appropriate modeling (simple direct effect model). That is, the urinary excretion of torsemide over time (as PKs) was linked to the urinary sodium excretion (as PDs). Through the substitution of model parameters, torsemide PK and PD outcomes were simulated according to different CYP2C9 phenotypes in healthy adult populations. Torsemide PK and PD simulations for CKD, liver cirrhosis, and CHF patient groups were performed by reflecting physiological and biochemical changes according to each disease group in model parameters. The structure of the PBPK-PD model was the same for both healthy adult and patient populations. Figure 1 presents the workflow of torsemide PBPK-PD modeling performed in this study.

### 2.3. PBPK-PD Model Construction

**Figure 2:**
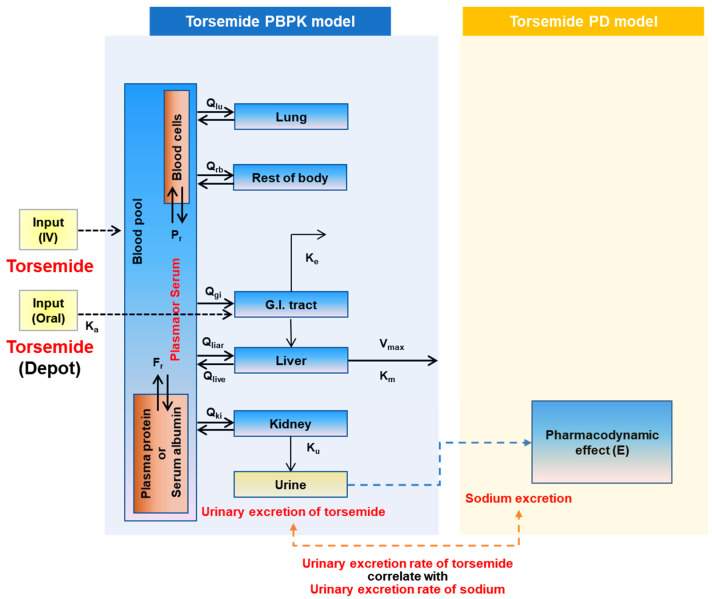
Structure of the torsemide PBPK-PD model.

The Torsemide PBPK-PD model structure was established based on previously reported pharmacological and pharmacokinetic information on torsemide [1,3,4]. Oral and intravenous routes of administration of torsemide are used in clinical practice [1]. Therefore, both routes of administration were reflected in the model. In the PBPK model, the liver, the main metabolic organ of torsemide, and the kidney, the excretory organ, were reflected as separate compartments. In addition, the gastrointestinal (GI)-tract compartment was reflected as a torsemide absorption pathway in the body following oral administration, and the lung was reflected in the model as a pulmonary circulation organ through which total blood would flow in and out. Other organs were considered as the rest of the body. Previous studies [9,10,11,12,13,14] have reported a significant correlation between the rate of torsemide excretion in urine and the urinary sodium excretion rate. Therefore, the association between the rate of excretion of torsemide into the urine via the kidneys and the rate of sodium excretion was established as a PD-model. According to previous reports [1,8], plasma protein (or serum albumin) binding efficiency of torsemide was as high as >90%, and torsemide could be distributed into blood cells. Therefore, the degree of plasma protein binding and distribution of torsemide to blood cells were reflected in the structural model. Figure 2 presents the PBPK-PD model structure of torsemide established in this study. Modeling and simulations were performed using Berkeley Madonna (Berkeley, CA, USA), S-ADAPT (USC, CA, USA), and Phoenix WinNonlin (Certara Inc., Princeton, NJ, USA).

### 2.4. PBPK-PD Model Parameterization

Torsemide movement between each tissue compartment was linked to blood flow rate and parameterized by Q. That is, Q_bl_, Q_ki_, Q_gi_, Q_rb_, Q_lu_, Q_live_, and Q_liar_ meant blood flow rates in the blood, kidneys, GI-tract, the rest of the body, lungs, hepatic vein, and hepatic artery, respectively. The volume of each tissue compartment was parameterized as V. V_bl_, V_ki_, V_li_, V_gi_, V_rb_, and V_lu_ meant volumes of blood, kidneys, liver, GI-tract, rest of the body, and lungs, respectively. The blood flow rate to each tissue and the volume value of each tissue used previously reported physiological values in humans [27,28,29,30]. Torsemide metabolism in the liver was reflected in a nonlinear saturation model based on past reports [4,6]. V_max_ and K_m_ were applied as related constants. V_max_ was the maximum metabolic rate of torsemide and K_m_ was the torsemide concentration at half the maximum metabolic rate. Although the in vivo PK of torsemide was linear at doses up to 200 mg [1], the in-vitro metabolism test confirmed nonlinearity, suggesting that there was a saturation interval in torsemide metabolism [4,6]. The nonlinearity of the invitro metabolism test was probably due to exposure to torsemide concentrations up to approximately 60 μM (high concentrations that are difficult to appear in vivo in clinical doses of torsemide) invitro. K_a_ was reflected as an absorption rate constant following oral administration. K_e_ was considered in relation to the loss of some torsemide from the GI-tract. K_u_ was reflected as the rate constant associated with torsemide excretion into the urine via the kidneys. K_a_, K_e_, and K_u_ were considered as first-order rate constants based on torsemide’s general PK-linearity [1]. F_r_ was incorporated as a model parameter as the unbound fraction of torsemide in plasma or serum (associated with binding to plasma proteins or serum albumin). P_r_ was parameterized as the partition ratio of torsemide from plasma to blood cells. Experimental invitro values related to V_max_, K_m_, F_r_, and P_r_ reported in previous studies [4,8] were applied as parameter values of the model. V_max_ and K_m_ were in vitro experimental values obtained using human liver microsome. F_r_ was the in vitro value obtained by the equilibrium dialysis method for human serum albumin. P_r_ had no values reported for humans. It was measured in vitro in rat blood. Other physiological or biochemical parameter values (such as V_rb_, Q_rb_, K_a_, K_u_, and K_e_) were estimated by fitting between observational data and model simulations. Partition coefficient (as K_p_) related to the distribution of torsemide from the blood to each tissue (kidney, liver, GI-tract, rest of body, and lung) was parameterized as K (K_ki_, K_li_, K_gi_, K_rb_, and K_lu_), with their values predicted by Simcyp^TM^ PBPK Simulator (version 21, Certara Inc., Sheffield, UK) based on physicochemical properties of torsemide. Quantitative physicochemical values of torsemide (such as molecular weight, log P, and pK_a_) derived from past studies and literature [1,31] were used. Information on parameters applied for torsemide PBPK-PD modeling and basic physicochemical information on torsemide are presented in Tables S2 and S3, respectively. Structural formulas of the model are presented in the Appendix A.

In the formula of PD-model (baseline sigmoid E_max_), E and x denoted rates of excretion of sodium and torsemide through the urine, respectively. E_0_ and E_max_ were basal and maximal rates of urinary sodium excretion, respectively. EC_50_ was the urinary excretion rate of torsemide equal to 50% of the maximum urinary excretion rate of sodium. P was reflected as the slope constant of the graph.

### 2.5. PBPK-PD Model Validation

The predictive power of the torsemide PBPK-PD model was verified using various tools. To verify that the model could explain multiple observations well overall, torsemide PK and PD observations from healthy adults (including CYP2C9 genetic polymorphisms) and various patient groups (CKD, cirrhosis, and CHF) were visually and numerically compared with model simulation results. Applied tools included a visual consistency check, root mean squared error (RMSE) check, Akaike information criterion (AIC) check, and a two-fold error check [32]. In addition, a sensitivity test was performed to confirm the appropriateness of parameters in the model structuring. To check the two-fold error, the area under the curve (AUC_0–∞_) and the maximum plasma concentration (C_max_) were calculated and determined based on time-plasma (or serum) torsemide concentration profiles. AUC_0–∞_ was estimated based on the trapezoidal rule.

### 2.6. PBPK-PD Model Application

The torsemide PBPK-PD model established based on data sets of healthy adult populations was extended to torsemide PK and PD simulations in healthy adult populations according to CYP2C9 genetic polymorphisms and in patient populations of cirrhosis, CKD, and CHF.

#### 2.6.1. PK and PD Simulations of Torsemide According to Different CYP2C9 Phenotypes

According to previous reports [4,5,6], the metabolism of torsemide in the body is mainly caused by the CYP2C9 enzyme present in the liver. Therefore, torsemide PK-diversity according to CYP2C9 genetic polymorphisms has a reasonable causal relationship. Related results have been reported in previous studies [5,7]. In this study, some healthy adult populations were divided into extensive-metabolizers (such as *1*1; EM), intermediate-metabolizers (such as *1*3; IM), and poor-metabolizers (such as *3*3; PM) for torsemide according to CYP2C9 genetic polymorphisms. Simulations of torsemide PKs and PDs for different CYP2C9 phenotypes were performed through changes in torsemide metabolic rate constants. That is, simulations were performed by changing the degree of intrinsic clearance of torsemide in EM, IM, and PM, respectively. The degree of decrease in torsemide metabolism according to different CYP2C9 phenotypes reflected previously reported results [6,33]. The reduction ratio of intrinsic clearance from EM to IM to PM was approximately 0.15. This was because V_max_ decreased but K_m_ increased from CYP2C9*1 to *3 in the enzyme kinetics of torsemide reported in the past [6,33]. As a result, intrinsic clearance tended to decrease (from CYP2C9*1 to *3). The degree was approximately 0.15. Similar to this study, in previous modeling studies [34,35], the prediction of the PK-profile of a substrate drug according to CYP2C9 genetic polymorphisms was performed by changing the metabolic rate constant. Table 1 presents parameter information applied to simulate torsemide PKs and PDs according to different CYP2C9 phenotypes.

#### 2.6.2. Prediction of Torsemide PKs and PDs in Cirrhosis Patients

Prediction of torsemide PKs and PDs for cirrhosis patients was performed through parametric substitutions of the established PBPK-PD model for healthy adults. This is because cirrhosis causes physiological and biochemical changes that are different from those in healthy adult populations, and these need to be reflected as independent variables in the PBPK-PD model. Liver cirrhosis is a disease caused by chronic hepatocyte damage. It affects the volume change of the liver and expression levels of plasma proteins and drug-metabolizing enzymes synthesized in the liver [36]. Therefore, cirrhosis not only causes changes in the blood flow rate to the liver and various tissues throughout the body but also changes body distribution and metabolism of drugs mainly metabolized by the liver [36]. Substitution of parameters was performed by reflecting physiological and biochemical changes caused by cirrhosis with reference to past reports [37,38,39,40]. Major physiological factors changed in cirrhosis patient populations were the volume of the liver and blood flow rates to tissues. Major biochemical change factors included plasma protein binding and distribution to blood cells (of torsemide), K_p_ to various tissues (of torsemide), decreased metabolism in the GI-tract (of torsemide), and decreased expression of metabolic enzymes in the liver. This was thought to be due to a significant decrease in plasma protein (such as albumin and α1-acid glycoprotein) concentration and hematocrit due to cirrhosis [41], resulting in possible changes in several physiological and biochemical parameters. The severity of cirrhosis was classified into A, B, and C according to the Child-Pugh (CP) classification commonly used in clinical practice [42]. Changes in physiological and biochemical parameters for each CP-A, B, and C group were reflected in values derived from past studies [37,38,39,40] and the ‘Sim-Cirrhosis CP-A, B, C’ group data of Simcyp^TM^ PBPK Simulator. F_r_, P_r_, and V_max_ were parameterized reflecting decreases in total protein, hematocrit, and CYP2C9 expression, respectively. V_max_ values in cirrhosis patient groups were estimated by multiplying healthy V_max_ by the ratio of the CYP2C9 expression level in the healthy and each cirrhosis patient group (based on the general assumption that expression levels of CYP enzymes tend to be proportional to V_max_). K_e_ was parameterized to reflect the increase in villous blood flow as the product of healthy K_e_ value and villous blood flow ratio of healthy and each cirrhosis patient group [40]. K_p_ for each tissue was parameterized with predicted values reflecting changed F_r_ and P_r_ in liver cirrhosis patient groups. Here, F_r_ and P_r_ for each cirrhosis patient group were estimated with the following equations: F_r, cirrhosis_ = 1/[1 + (1 − F_r, healthy_) × PT_cirrhosis_/(PT_healthy_ × F_r, healthy_)] and P_r, cirrhosis_ = P_r, healthy_ × hematocrit ratio of healthy and each cirrhosis patient group [43]. In the formula, F_r, cirrhosis,_ and P_r, cirrhosis_ were values of F_r_ and P_r_ in each cirrhosis patient group (as CP-A, B, and C). F_r, healthy,_ and P_r, healthy_ were values of F_r_ and P_r_ in the healthy group. PT_cirrhosis_ and PT_healthy_ were total protein (as albumin and α_1_-acid glycoprotein) concentrations in cirrhosis patient groups and the healthy group, respectively. Changes in physiological and biochemical parameters applied in past studies [37,38,39,40] to predict drug PKs in cirrhosis patient groups were similarly reflected in this study. Table 2 presents parameter information applied to simulate torsemide PKs and PDs according to the severity of cirrhosis.

#### 2.6.3. Prediction of Torsemide PKs in CKD Patients

Prediction of torsemide PKs for CKD patient populations was performed through parametric substitutions of established PBPK models for healthy adults. CKD refers to a disease in which chronic kidney damage and resulting blood filtration performance are reduced compared to those of healthy populations [44]. Therefore, CKD can cause changes not only in the volume of the kidney but also in the blood flow to the kidney. As a result, it affects the degree of drugs excreted in the urine [44]. Substitution of parameters was performed by reflecting physiological and biochemical changes according to CKD with reference to past reports [45,46,47] and the ‘Sim-Renal GFR 30–60 and Sim-Renal GFR less 30’ group data of Simcyp^TM^ PBPK Simulator. Major physiological factors that changed in CKD patient populations were renal volume and blood flow to the kidneys. Major biochemical change factors were reduction of metabolic rate in the liver and rate constant of excretion (of torsemide) through urine. It was thought that CKD could lead to changes in renal and non-renal (as hepatic) clearance possibly due to a significant decrease in blood flow to kidneys [45,46,47]. Previous reports [45,46,47] have shown that renal disease is closely related to the deterioration of liver function. When the severity of renal disease is increased, liver function and hepatic clearance are decreased. Perhaps, as kidney disease worsens, the accumulation of waste products such as uremic solutes in the body increases, which might interfere with the functions of enzymes and various transporters. The severity of CKD can be classified according to the glomerular filtration rate (GFR) level, which is a commonly accepted classification in clinical practice [48]. Changes in physiological and biochemical parameters for each CKD group were reflected in values derived from past studies [45,46,47]. Changes in physiological and biochemical parameters applied in past studies [45,46,47] to predict drug PKs in CKD patient groups were similarly reflected in this study. As CKD severity increased, blood flow to kidneys decreased. Such decrease was compensated for by the rest of the body. This was a setting that assumed the existence of a compensatory mechanism (such as renin-angiotensin-aldosterone system) in the body as much as the degree of decrease in renal blood flow as well as mass balance, which was generally assumed in a PBPK model at a situation where quantitative information on the decrease or increase in cardiac output in the CKD patient groups (especially for each CKD stage) was lacking. Liver intrinsic clearance was V_max_/K_m_. V_max_ values for the severity in CKD patient groups were adjusted to match reported liver intrinsic clearance fractions. Table 3 presents parameter information applied to simulate torsemide PKs according to CKD severity.

#### 2.6.4. Prediction of Torsemide PKs and PDs in CHF Patients

Prediction of torsemide PKs and PDs for CHF patient populations was performed through parametric substitutions of established PBPK models for healthy adults. CHF refers to a disease condition in which the heart cannot pump blood effectively [49]. Therefore, CHF directly affects systemic blood flow rates. In the case of drugs metabolized in the liver, CHF also affects the degree of liver-metabolism along with a decrease in the hepatic blood flow rate [49]. Parameters were replaced by reflecting physiological and biochemical changes according to CHF with reference to previous reports [50,51]. The main physiologic and biochemical factors altered in the CHF patient groups were blood flow rate to various tissues and reductions of metabolism in the liver, respectively. It was thought that CHF caused changes in hepatic clearance due to a significant decrease in blood flow to various tissues. The severity of CHF was classified as mild, moderate, and severe based on the New York Heart Association (NYHA) classification generally applied in clinical practice [52]. The degree of decrease in V_max_ according to the severity of CHF was reflected in consideration of the decrease in blood flow to the liver [50,51]. That is, the V_max_ for each CHF patient group was estimated by multiplying the V_max_ for the healthy group by the fraction of blood flow to the liver of each CHF patient group. This was based on the fact that in CHF patient groups, the degree of hepatic-metabolism was affected by the rate of blood flow to the liver [53]. A decrease in hepatic clearance in CHF patient groups might be related to a decrease in the activity of enzymes expressed in the liver due to decreased blood supply to the liver. Q_lu_ reflected results according to overall blood flow rate change in CHF patient groups. Q_live_ reflected results of Q_liar_ and Q_gi_ changes in CHF patient groups. Table 4 presents information on parameters applied to simulate torsemide PKs and PDs according to CHF severity.

## 3. Results and Discussion

### 3.1. Establishment of the Torsemide PD Model

For torsemide PDs, the relationship between the urinary excretion rate of torsemide and the urinary sodium excretion rate according to torsemide exposure was applied. According to past reports [9,10,11,12,13,14,15] of torsemide PD-data, urinary sodium excretion rates were mainly related to urinary torsemide excretion rates. Since the main mechanism of action of torsemide, a diuretic, is excreting sodium through urine [1], the rate of sodium excretion in urine was sufficient to be applied as PD-data for torsemide. PDs of torsemide in healthy adult populations, cirrhosis, and CHF patients were all explained by a baseline sigmoid E_max_ model. Table 5 presents parameter values and equations of the torsemide PD-model for healthy adults, cirrhosis, and CHF patients. Regarding CKD, PD-reporting data for torsemide could not be definitively confirmed. Interestingly, there were significant differences in the efficacy and potency of torsemide PDs in healthy adult populations, cirrhosis patients, and CHF patients. The E_max_ of the cirrhotic group was 1.61 times lower than that of the healthy adult group. The EC_50_ of the CHF patient group was approximately 11.53 times higher than that of the healthy adult group. The reason for such differences in efficacy and potency of torsemide PDs between groups was thought to be physiological or biochemical changes caused by the disease. For example, the activity or function of the Na^+^/K^+^/2Cl^−^ cotransporter located in the ascending loop of Henle, the target of torsemide, might be affected by the disease. It has already been confirmed that the expression of Na^+^/K^+^/2Cl^−^ cotransporter is reduced in the ascending loop of Henle in animal models (liver cirrhosis rats) [54]. It has been suggested that changes in the activity and expression of Na^+^/K^+^/2Cl^−^ cotransporter occur in humans with edematous disorders [55]. In addition, there have been reports that changes in enzyme expression and functions are caused by various diseases [37,38,45,46]. The result that the E_max_ value in the cirrhosis patient group was 1.61 times lower than that of the healthy adult group indirectly suggested that the PD-effect (on torsemide) of decreased renal blood flow and transporter (Na^+^/K^+^/2Cl^−^ cotransporter) function (with expression) was relatively larger than that of CYP2C9 genetic polymorphism. The large increase in EC_50_ value (11.53 times larger than the healthy adult group) compared to the decrease in E_max_ in the CHF patient group (a mild decrease of 1.26 times compared to the healthy adult group) suggested that the expression of the transporter was not significantly affected, although the affinity [with the substrate (torsemide)] was significantly decreased. Although clear evidence is lacking, this might be related to a decrease in the interaction between the transporter and torsemide due to increases in endogenous transporter inhibitors in CHF patient groups. For example, it has been reported that endogenous nitric oxide synthase inhibitors are increased in CHF patients [56]. Therefore, in CHF, endogenous antagonists for enzymes and/or various transporters may be increased due to abnormal blood circulation and pathological actions, and these may be factors that interfere with the interaction between transporters and substances. A PD-model for the CHF patient group was established based on PD results obtained from groups presumed to have a mild to moderate disease (NYHA Class II or III) [13,26]. A PD-model for the cirrhosis patient group was established based on PD results obtained from groups presumed to have CP-B cirrhosis [10]. In each group, the urinary sodium excretion rate according to the urinary torsemide excretion rate had a sigmoidal or curved pattern. The correlation coefficient values of the model were all 0.81 or more (90% correlation). AIC values were 63.12–173.8. These results suggested that the PD-model for healthy adults, cirrhosis patients, and CHF patients could explain torsemide PD observations well and reliably predict PDs of torsemide. Figure S1 presents fitting results of the torsemide PD-model for healthy adults, cirrhosis patients, and CHF patients.

### 3.2. PBPK-PD Modeling for Healthy Adult Populations

A previous report [1] has confirmed that PK-linearity exists with torsemide up to a dose of at least 200 mg. However, it has also been reported that the metabolism of torsemide follows Michaelis–Menten kinetics in invitro tests using human liver microsome [4,6]. Related results were reflected in this torsemide PBPK model. This could also secure applicability to a wider range of torsemide dosages through the extension of the model. Model simulation results confirmed that the PBPK model established in this study exhibited PK-linearity at torsemide doses up to 200 mg or more. Torsemide K_p_ values for each tissue in healthy adult populations predicted by the Simcyp^TM^ PBPK Simulator were as low as 1 or less. Although there might be differences between species, a past study in rats showed that torsemide K_p_ values in several tissues were lower than 1 [8], consistent with the Simcyp^TM^ PBPK Simulator prediction. That is, the predicted pattern of torsemide K_p_ for each tissue in humans was not largely different from its experimental distribution results in rats [8]. Figure S2 shows a comparison between torsemide K_p_ values in a healthy adult population (predicted) and reported values in rats [8] (observed).

Plasma or serum concentrations of torsemide following a single oral or intravenous exposure to torsemide observed in healthy adult populations [9,10,11,16,17,18,19,20,21] were well-fitted by the PBPK model. It was confirmed that torsemide plasma or serum concentrations according to torsemide dose (5, 10, and 20 mg) in each administration route were fitted relatively well by the model. That is, most of the observed individual values or average values were evenly distributed up and down without a large error based on the average value of the model simulation. Figure S3 presents the plasma or serum torsemide concentration profile and model simulation results according to oral or intravenous exposure to torsemide at doses of 5, 10, and 20 mg. It was confirmed that 72–84 h plasma torsemide concentration results in healthy adults obtained after multiple oral administrations at 24 h intervals [18] were well-fitted by the established torsemide PBPK model. That is, most of the observed mean values were within 95% confidence intervals of the model simulation. Figure S4 shows plasma concentration values observed after multiple oral exposures to 10 mg torsemide and simulation results by the model.

Cumulative urinary excretion of torsemide following a single oral or intravenous exposure of torsemide observed in healthy adult populations [9,10,19] was well-simulated by the PBPK model. That is, most of the observed cumulative torsemide urine excretion average values were distributed without large errors in the average values of the model simulation. The cumulative excretion amount of torsemide in the urine observed after a single oral or intravenous exposure to 10 or 20 mg of torsemide and simulation results by the model are presented in Figure S5. Results shown in Figures S3–S5 suggest that the model established in this study can capture PKs (of torsemide) in plasma and urine following single or multiple exposures to torsemide at different doses in healthy adult populations. Urinary sodium excretion rates following a single oral or intravenous exposure to torsemide observed in healthy adult populations [10] were well simulated by the PBPK-PD model. That is, most of the average urinary excretion rate values of sodium observed after oral or intravenous administration of 10 mg torsemide were distributed without significant error in the average value of model simulation (according to exposure to 10 mg torsemide). This suggested that the PBPK-PD model established in this study could well capture urinary PDs following a single exposure to torsemide in healthy adult populations. Figure S6 presents values of the sodium excretion rate in the urine after a single oral or intravenous exposure to torsemide and simulation results by the model.

From these results, the following conclusions could be made. First, the PBPK-PD model proposed in this study could well describe plasma (or serum) torsemide concentration patterns in healthy adult populations, which have been independently conducted in several studies (at doses of 5, 10, and 20 mg through intravenous or oral route by single or multiple administrations). Second, the pattern of cumulative excretion of torsemide in the urine and the rate of excretion of sodium in the urine, which is an index for evaluating the efficacy of torsemide, can be well explained by the PBPK-PD model. These results suggest that the torsemide PBPK-PD model constructed and presented in this study is a universal model that well explains changes in urinary sodium excretion rates along with torsemide plasma (or serum) concentration and urinary excretion patterns in healthy adults.

### 3.3. PBPK-PD Model Simulation According to Different CYP2C9 Phenotypes

Simulations of torsemide PKs and PDs according to different CYP2C9 phenotypes were performed to reflect differences in liver-metabolism based on an established PBPK-PD model in healthy adults. A previous study [7] has shown that genetic polymorphisms of CYP2C9 and OATP1B1 (organic anion transporting polypeptide 1B1) are involved in PK-diversities of torsemide within a population. That is, genetic polymorphisms of CYP2C9 and OATP1B1 in systemic clearance and distribution volume variability of torsemide, respectively, in healthy adult populations, were identified as effective covariates. Genetic polymorphisms of CYP2C9 and OATP1B1 had separate effects on the clearance and distribution of torsemide, respectively. However, it was confirmed that the effects of CYP2C9 genetic polymorphisms on PK-diversities of torsemide were greater than those of OATP1B1. The mean dose-normalized AUC_0–∞_ variation according to different CYP2C9 phenotypes (EM versus IM in the same OATP1B1 phenotype) was 28.43–34.01%. The degree of variation was statistically significant (Student’s *t*-test, *p* < 0.05). Mean dose-normalized AUC_0–∞_ variations according to different OATP1B1 phenotypes [extensive-transporter (ET) versus intermediate-transporter (IT) versus poor-transporter (PT), in the same CYP2C9 phenotype] were 0.35–3.47%. These variations were not statistically significant (Student’s *t*-test or ANOVA, *p* > 0.05). Proportions of CYP2C9 and OATP1B1 in overall torsemide PK-variabilities (estimated based on the AUC_0–∞_ value) were approximately 90–99% and 1–10%, respectively. Considering that potential covariates might not have been explored yet and PK-variability between individuals even within PK-differences due to different OATP1B1 phenotypes, the effect of OATP1B1 on torsemide PK-diversity was expected to be significantly lower than that of CYP2C9. Figure S7 shows comparison results of dose-normalized AUC_0-∞_ according to a phenotypic combination of CYP2C9 and OATP1B1. As a result, in this PBPK modeling study, model simulations were mainly performed according to genetic polymorphisms of CYP2C9. Results according to CYP2C9 phenotypes were relatively more clearly accessible than quantitative reports on differences in transporter expression and activity according to OATP1B1 phenotypes. Since the in vivo metabolism of torsemide is mainly due to CYP2C9 [1,4,5,6], torsemide PK-diversities according to genetic polymorphisms of CYP2C9 [5] are relatively more important than other genetic polymorphisms. Obviously, the PBPK-PD model developed in this study did not imply that OATP1B1 was not involved in torsemide PK-diversity, but rather an attempt to simulate torsemide PKs and PDs according to polymorphisms of CYP2C9 that had a greater effect on torsemide PK-diversity than those of OATP1B1. In the future, if degrees of influence of OATP1B1 on PK and PD diversity of torsemide are confirmed for more individuals, especially if results regarding the activity (related to the transport of the substrate torsemide) and expression level according to different OATP1B1 phenotypes are obtained, they can be applied to this PBPK-PD model.

Plasma or serum concentrations of torsemide for each CYP2C9 phenotype [5] were well-fitted overall by the PBPK model. Plasma or serum concentrations of torsemide slightly increased from EM to IM to PM within the same dose group. These trends were well captured by the mean values of the model simulation. Figure S8 shows plasma or serum torsemide concentrations observed according to different CYP2C9 phenotypes after a single oral exposure to torsemide and simulation results by the model.

The cumulative urinary excretion amount of torsemide and urinary sodium excretion rate profiles according to the CYP2C9 phenotypes were simulated by the established PBPK-PD model. It was confirmed that the cumulative urine excretion amount of torsemide increased from EM to IM and PM. The urinary sodium excretion rate also increased. This might be related to the increase in values from EM to IM to PM in the plasma or serum concentration profile of torsemide. That is, from EM to IM and PM, the metabolism of torsemide in the body decreased. Thus, the unaltered torsemide in plasma or serum would have increased. This might have resulted in increased excretion of torsemide and sodium into the urine. Figure S9 presents model simulation results of torsemide accumulation and sodium excretion rate in the urine according to different CYP2C9 phenotypes after a single oral exposure to 10 mg of torsemide. As a result, it was found that the PBPK-PD model of torsemide constructed and presented in this study could sufficiently explain PKs and PDs of torsemide according to genetic polymorphisms of CYP2C9.

### 3.4. Simulation of the PBPK-PD Model for Cirrhosis Patient Groups

To simulate torsemide PK and PD patterns in cirrhotic patient populations, physiological and biochemical changes following cirrhosis were reflected in an established model for healthy adult populations. Figure S10 shows parameter changes reflected according to the severity of cirrhosis. All other parameters such as V_bl_, V_lu_, V_ki_, V_gi_, V_rb_, K_m_, K_a_, and K_u_ were reflected the same as values applied to healthy adult groups (Table S2). Plasma concentrations and cumulative urinary excretion of torsemide in cirrhosis patient groups [10] were fitted relatively well by the PBPK model reflecting physiological and biochemical changes in cirrhosis patient groups. Plasma torsemide concentration profiles and cumulative urine excretion of torsemide differed between cirrhotic patient groups (such as CP-A, CP-B, and CP-C). From CP-A to CP-B and CP-C, it was confirmed that torsemide in plasma decreased gradually with a lower terminal slope and continuously excreted through urine with a higher terminal slope. In addition, torsemide plasma concentration and cumulative urine excretion amount were predicted to be higher in cirrhotic patient groups than in healthy adult groups. This pattern is similar to the prediction that torsemide is metabolized mainly in the liver. Thus, the degree of its metabolism will vary depending on the degree of liver disease. However, the reason that the plasma concentration profile and urinary excretion of torsemide were lower in CP-B and CP-C than in CP-A might be related to the degree of large physiological changes according to the severity of liver cirrhosis. That is, from CP-A to CP-B and CP-C, V_li_ and V_max_ decreased by 34.57% and 52.18%, respectively (Figure S10). Blood flow rates to various tissues (such as Q_bl_, Q_lu_, Q_rb_, Q_live_, and Q_liar_) in the body except Q_ki_ and Q_gi_ were increased (11.34–40.86%). K_p_ to each tissue (such as K_lu_, K_ki_, K_li_, K_gi_, and K_rb_) was also increased (4.99–12.05%). F_r_ values were increased by 51.14% due to a decrease in total plasma protein associated with impaired hepatic protein synthesis. As a result, lower plasma concentration profiles in CP-B and CP-C than in CP-A suggested that the clearance of torsemide in cirrhotic patient groups was more likely due to the rate of blood flow, not the rate of intrinsic liver-metabolism. This could be explained by the extensive hepatic-metabolism of torsemide [1]. This is because the kinetics of drugs with a low hepatic extraction are more sensitive to liver failure than to changes in hepatic blood flow, whereas drugs with significant first-pass effects are sensitive to changes in hepatic blood flow [57]. Therefore, increasing blood flow rates to the liver and other tissues might have had a more significant effect on PKs of torsemide than decreased liver function from CP-A to CP-B and CP-C. For example, similar to this study, observed plasma concentrations of cabozantinib in cirrhosis patient groups were higher in mild (as CP-A) than in moderate (as CP-B) and the predicted cabozantinib plasma concentration profile in the model simulation was higher in mild than in moderate [58]. The interpretation of these results [58] was that the unbound fraction of cabozantinib increased in moderate than in mild. As a result, its clearance was increased in moderate than in mild. Thus, a low plasma concentration profile was predicted in moderate. It was also thought to be the result of a similar trend to blood flow rate metabolism as a whole.

Decreased urinary excretion of torsemide in CP-B and CP-C than in CP-A was associated with a 20.55% decrease in Q_ki_ from CP-A to CP-B and CP-C. Figure S11 shows torsemide concentrations in plasma and accumulated urinary excretion (of torsemide) according to the severity of cirrhosis after a single oral or intravenous exposure to 10 mg of torsemide and simulation results by the model. Past studies [37,38,39,40] have reported that cirrhosis patients may differ in several biochemical factors compared to healthy adults. Therefore, for the prediction of torsemide PKs and PDs in cirrhosis patient groups, changes in values of many model parameters were reflected (Figure S10). Even within cirrhosis patient groups, the degree of change in parameter values according to the severity (CP-A, CP-B, and CP-C) was large. In particular, ranges of changes in total blood flow, blood flow rate to each tissue, and liver volume were significant. In addition, changes in plasma protein binding and plasma-blood cell partition ratio of torsemide due to a decrease in protein synthesis caused by liver disease were reflected in the model. Thus, PK and PD patterns of cirrhosis patient groups were different from those of healthy adult groups.

Urinary sodium excretion rates observed in the CP-B cirrhosis group after torsemide administration [10] were well-simulated by the PBPK-PD model reflecting physiological and biochemical changes in cirrhotic groups. Figure S12 shows the rate of sodium excretion in the urine observed after a single oral or intravenous exposure to 10 mg torsemide in CP-B patients and simulation results by the model. Predicted values of the urinary sodium excretion rate at approximately 4–6 h after torsemide administration were close to baseline E_o_ values in healthy adults (4.39 mEq/h) and cirrhosis groups (4.33 mEq/h). This suggests that the maximal diuretic effect of torsemide in the CP-B patient group would appear within 4 h approximately, after administration. Predicted urinary sodium excretion rate profiles for single oral or intravenous exposure to torsemide in CP-A and C patients are presented in Figure S13. After exposure to torsemide 10 mg, it was confirmed that the sodium excretion rate in the urine returned to the baseline (E_0_) value more rapidly as cirrhosis progressed from CP-A to CP-B and CP-C. In this study, the PD-model for cirrhosis patient groups was established based on PD-results obtained from patient groups presumed to have CP-B. Therefore, PDs for CP-A or CP-C patients were predicted based on the established PD-model for CP-B. In this study, through PBPK-PD modeling, PKs and PDs of torsemide could be predicted for patient groups that were difficult to conduct experimentally. It was also found that the presented torsemide PBPK-PD model could be used as a universal predictive model as it could well explain changes in the urinary sodium excretion rate along with torsemide plasma concentration and urinary excretion pattern in cirrhosis patients. However, in the future, through the experimental confirmation of torsemide PD-efficacy and potency for CP-A and CP-C, it will be possible to more clearly predict PDs in each cirrhosis patient group.

### 3.5. Simulation of the PBPK Model for CKD Patient Groups

To simulate torsemide PK-patterns in CKD patient populations, physiological and biochemical changes following CKD were reflected in an established model for healthy adult populations. Figure S14 shows parameter changes reflected according to CKD severity. All other parameters such as V_bl_, V_lu_, V_li_, V_gi_, V_rb_, Q_bl_, Q_lu_, Q_gi_, Q_live_, Q_liar_, K_lu_, K_ki_, K_li_, K_gi_, K_rb_, F_r_, P_r_, K_m_, K_a_, and K_e_ were reflected the same as values applied to healthy adults (Table S2). Plasma concentrations and accumulated urinary excretion of torsemide in CKD patient groups [24] were well-fitted by the PBPK model reflecting physiological and biochemical changes in CKD patient groups. Figure S15 shows plasma concentration and the accumulated urinary excretion of torsemide according to the severity of CKD after a single oral or intravenous exposure to 100 mg of torsemide and simulation results by the model. Differences between CKD patient groups (such as mild, moderate, and severe) in plasma torsemide concentration profiles were not significant. On the other hand, for cumulative excretion of torsemide in urine, it was confirmed that the difference between CKD groups was larger than the plasma concentration profile. That is, from mild CKD to moderate and severe, plasma torsemide concentration profiles were almost similar, whereas torsemide excretion in urine was significantly reduced. This was similar to a previous report showing that the degree of renal function had little effect on the total clearance of torsemide and that it was linearly related only to the renal clearance of torsemide [1].

PD-prediction for CKD patient populations will need to be conducted in the future based on experimental PD-data (related to urinary excretion) performed on CKD patient populations. There was a limit to simply applying the PD-model for healthy adults to CKD patient groups in a situation where clear experimental PD results for CKD patient groups were not confirmed. This was because there were relatively large differences in the efficacy and potency of PDs for cirrhosis and CHF patients than for healthy adults. Therefore, it was predicted that PDs in CKD patient groups, which was different from that of healthy adults, could be identified as well. Although torsemide PD-data for patients with moderate and severe CKD have been reported [14], their application as a torsemide PD-model for CKD patients in this study was limited. In the past report [14], PD-data obtained for CKD patient groups showed that the urinary torsemide excretion rate and sodium excretion rate continued to increase in a proportional (almost straight) relationship. This might be because sodium excretion rates were measured at relatively low rates of urinary torsemide excretion (with a maximum value of less than 400 μg/h). Therefore, sodium excretion rates at higher urinary torsemide excretion rates (such as 800–1000 μg/h) need to be measured. This is because there is a ceiling point (as maximum efficacy) in torsemide PDs (of CKD patient groups) when compared with torsemide PD-data collected for healthy adult populations and other patient groups (Figure S1). As a result, it was difficult to establish a suitable PD-model for CKD patient groups based on the current level of data alone [14].

### 3.6. Simulation of the PBPK-PD Model for CHF Patient Groups

To simulate torsemide PK-patterns in CHF patient populations, physiological and biochemical changes following CHF were reflected in an established model for healthy adult populations. Figure S16 shows parameter changes according to CHF severity. All other parameters such as V_bl_, V_lu_, V_ki_, V_li_, V_gi_, V_rb_, K_lu_, K_ki_, K_li_, K_gi_, K_rb_, F_r_, P_r_, K_m_, K_a_, K_u_, and K_e_ were reflected the same as their values applied to healthy adult groups (Table S2). Plasma concentrations and accumulated urinary excretion of torsemide in CHF patient groups [11,12,13,25,26] were well-fitted by the PBPK model reflecting physiological and biochemical changes in CHF patient groups. Figures S17 and S18 show concentrations in plasma and accumulated urine output of torsemide according to the severity of CHF after a single oral or intravenous exposure to 10–200 mg torsemide and simulation results by the model. Plasma concentration profiles and cumulative urinary excretion of torsemide differed between CHF patient groups (such as mild, moderate, and severe). From mild CHF to moderate and severe, it was confirmed that plasma torsemide concentrations were gradually decreased (according to exposure time). It was also confirmed that accumulated amounts of torsemide in the urine were higher in severe CHF than in mild or moderate. This might be related to a significant overall decrease in blood flow to tissues and a decrease in torsemide metabolism in the liver as the severity of CHF increases. However, as confirmed by observations and model simulation results, differences in PK between CHF groups at the same dose of torsemide were not very large from that in cirrhosis patient groups. This suggested that the severity of liver disease with changes in several physiological and biochemical parameters as in cirrhosis patients had a greater effect on PKs of torsemide.

PD results following oral administration of 50, 100, and 200 mg of torsemide observed in the CHF moderate group [13] were relatively well simulated by the PBPK-PD model reflecting physiological and biochemical changes in CHF patient groups. That is, it was confirmed that the rate of sodium excretion through urine increased as the torsemide dose increased in the CHF moderate patient group. These trends were well captured by the model. Figure S19 shows the rate of sodium excretion in the urine observed after a single oral exposure to 50–200 mg of torsemide in a patient group with moderate [13] or severe CHF [12] and simulation results by a model. Figure S19 shows the urinary sodium excretion rate observed in the severe CHF patient group following a single intravenous administration of 5–20 mg torsemide [15] and simulated results by the model. It was found that the torsemide PBPK-PD model presented in this study explained well the changes of torsemide PK/PD (plasma or serum concentration, urine excretion, and sodium excretion rate) in healthy adults with different CYP2C9 genotypes, cirrhosis patients, CKD patients, and CHF patients.

In this study, the PD-model for CHF patient groups was established based on PD results obtained from patients with CHF estimated to be moderate. Therefore, PDs for the mild or severe patient group was predicted based on the established PD-model for moderate CHF. The reason the model overestimated observations in PD for the CHF severe group [12,15] was probably due to the inconsistency of the PD-model. Actual PDs of torsemide for the CHF severe group might be different from that for the CHF moderate group. That is, it is likely that lower efficacy and potency will be observed in severe CHF than in moderate CHF. This was a predictable part based on the fact that there were differences in the efficacy and potency of torsemide PDs between healthy adult groups and other disease groups (such as cirrhosis and CHF). In the case of furosemide, an allogeneic diuretic drug of torsemide, it has been reported that there is a difference in PDs between healthy adult groups and patients with liver cirrhosis, CHF, and renal disease [59]. In most cases, responses to furosemide were lower in patient groups (even with regard to severity) than in healthy groups, suggesting that there were differences in efficacy and potency in PDs between groups. Therefore, it will be necessary to confirm the efficacy and potency of torsemide PDs in mild and severe CHF groups in the future.

### 3.7. Torsemide PK-PD Relationship

The newly constructed and proposed torsemide PK-related PD-model in this study is a simple direct-effect model. Torsemide PDs in healthy adults, cirrhosis, and CHF patients could be explained well by this model. The simple direct effect model of torsemide proposed in this study could be fully explained mechanistically based on the physiological system. As mentioned earlier, the pharmacological site of action of torsemide is directly related to the excretion of torsemide through urine. That is, an increase in the excretion of torsemide through the urine will lead to a simultaneous increase in the pharmacological response at the site of torsemide PD-action. Consequently, torsemide PK and PD actions occur as simultaneous responses at the same site (ascending loop of Henle). Thus, the direct response model could be explained reasonably. Although furosemide PD, an allogeneic diuretic drug, was described as an indirect response model [60], torsemide was explained by a direct response model due to its high potency. That is, the affinity for the same receptor (Na^+^/K^+^/2Cl^−^ cotransporter) and potency of torsemide were approximately 2–4 times higher than those of furosemide [1]. Therefore, unlike furosemide, torsemide would have been able to have a direct response due to its high potency and receptor affinity.

To confirm the relationship between torsemide PKs and PDs, the simulation results of torsemide plasma concentration and urinary excretion rate, and urine sodium excretion rate at the same time point were plotted (three-dimensionally or two-dimensionally). As a result, counterclockwise hysteresis was confirmed after oral administration of torsemide in healthy adult groups, cirrhosis patients, and CHF patients [in the three-dimensional plot and the two-dimensional plot between the urinary sodium excretion rate and torsemide plasma (or serum) concentration]. This might be related to the delay in the distribution from the initial absorption phase to the drug effect compartment after oral administration of torsemide. On the other hand, when torsemide was administered intravenously, there was no significant time difference between torsemide concentration and efficacy in healthy adults, cirrhosis patients, or CHF patients. These results are related to the direct transfer of torsemide to the site of action without a separate absorption process for intravenous administration (as bolus), unlike oral administration. Two-dimensional plots between the torsemide urinary excretion rate and urinary sodium excretion rate did not reveal any major time difference in healthy adult populations, cirrhosis patients, or CHF patients, regardless of the route of administration. As a result, considering the relationship between PKs and PDs of torsemide comprehensively, it was determined that the PK-PD relationship was closer to an instantaneous response (as a simple direct effect model) that showed an immediate effect than a delayed response. Therefore, it could be inferred that torsemide transferred into the blood was transported to the target very quickly to exert its drug effect. Figure S20 presents the results of the analysis of the association between torsemide PKs and PDs established in healthy adult groups, cirrhosis, and CHF patients.

### 3.8. Model Validation

As a result of the visual consistency check, it was confirmed that all observed torsemide plasma concentrations, urinary torsemide cumulative excretion amounts, and urinary sodium excretion rate values were in good agreement with values predicted by the PBPK-PD model. The developed torsemide PBPK-PD model was validated by a two-fold error, a quantitative comparison tool between simulated and observed values. As a result, all simulated values for torsemide plasma (or serum) AUC_0–∞_ and C_max_ and urine torsemide cumulative excretion values showed a difference within two-fold from the observed values. With regard to PDs, simulated values of sodium excretion rate in urine over time after torsemide administration showed a difference within two-fold from observed values. Two-fold error values were estimated as ratios between parameters or outcomes predicted by the model and parameters or outcomes calculated by observed values. Final two-fold error values were considered suitable only if they were between 0.5 and 2. As a result of checking the RMSE for judging the model fit using the residual degree between each individual observation value and the model prediction values, all values of torsemide PKs and PDs were below 100. As a result of the AIC check known to judge model fit by comprehensively considering the complexity and performance (difference from individual predicted values to observed values) of the model, all values of torsemide PKs and PDs were below 200. These model validation results suggested that the PBPK-PD model of torsemide established in this study well-fitted the observations overall. As a result of the normalized model sensitivity test for checking the range of change in the simulation result according to the change of model parameters, sensitivity values for AUC_0–∞_ and C_max_ were all between +1 and −1, confirming that the normalized model sensitivity did not change according to the dose of torsemide. This suggested that parameters applied to the torsemide PBPK model established in this study were reflected at an appropriate level without being heavily biased on specific factors. Figure S21 presents the parametric sensitivity test results of the torsemide PBPK model.

As a result, it was found that the presented torsemide PBPK-PD model had characteristics as a universal model that could well explain changes in torsemide PKs/PDs (plasma or serum concentration and urinary cumulative excretion of torsemide and sodium excretion rate according to torsemide doses, single or repeated administration, intravenous or oral administration) in various population groups (healthy adults with CYP2C9 genetic polymorphisms, cirrhosis patients, CKD patients, and CHF patients). In this study, based on the PBPK-PD model of torsemide, we tried to suggest a precise drug therapy for CYP2C9 genetic polymorphisms and each disease such as cirrhosis, CKD, and CHF.

### 3.9. Model Simulation by Scenario for Various Groups

The torsemide PBPK-PD model established based on clinical data was used to simulate PKs and PDs (of torsemide) in healthy adult populations with CYP2C9 genetic polymorphisms, CKD patients, cirrhosis patients, and CHF patients according to torsemide dosing intervals or doses. Model simulation results confirmed that the average plasma (or serum) concentration at steady-state and PDs (as the rate of sodium excretion through urine) increased by approximately three times when the dosing interval became shorter (from 12 h to 4 h) after torsemide was administered at multiple doses. In addition, it was confirmed that the difference between the minimum and maximum urinary sodium excretion rates (12.08–75.68 at 12 h and 66.97–95.83 at 4 h) at steady-state decreased by approximately two times as the torsemide dosing interval became shorter. This suggested that urinary sodium excretion could be increased by increasing torsemide dosing frequency in healthy adult populations.

**Figure 3:**
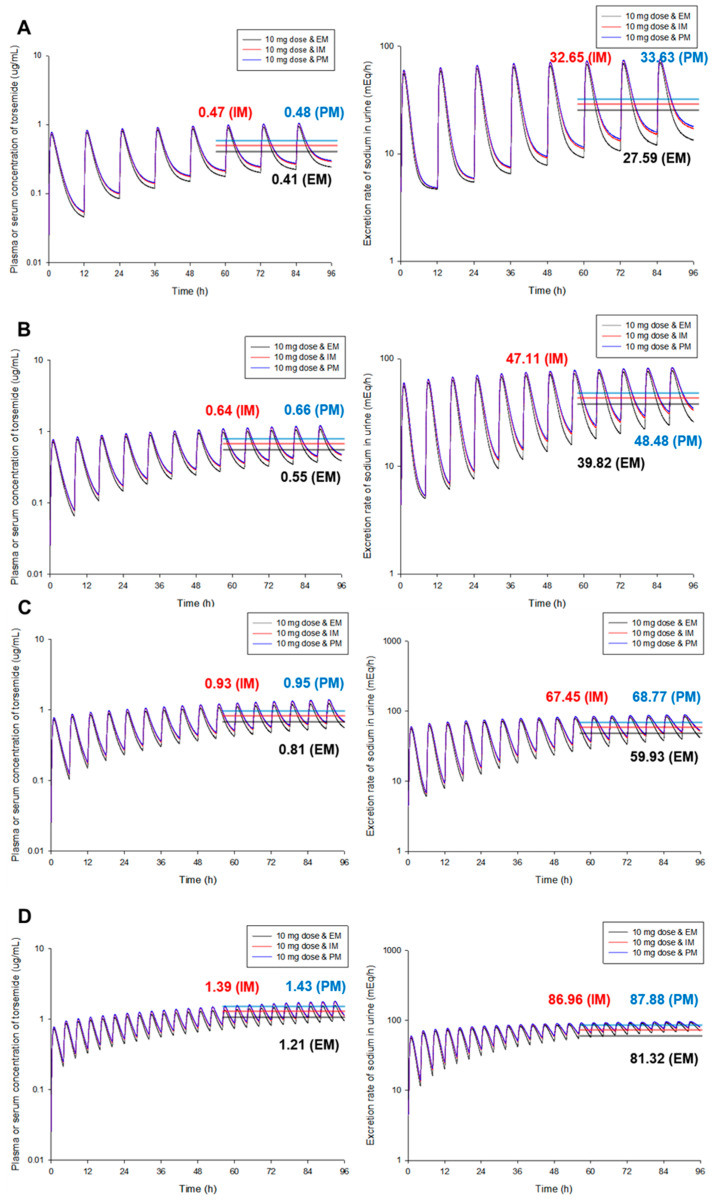
Simulation of plasma (or serum) torsemide concentration (left) and urinary sodium excretion rate (right) following oral multiple exposures at (**A**) 12, (**B**) 8, (**C**) 6, and (**D**) 4 h dosing intervals of 10 mg torsemide (according to different CYP2C9 phenotypes in healthy adult populations). The line presented parallel to the X-axis means values in the steady state.

Figure 3 shows PK and PD simulation results at steady-state according to changes in torsemide dosing interval for each CYP2C9 phenotype. As with single exposure to torsemide, it was confirmed that steady-state concentration of torsemide in plasma (or serum) increased as it changed to EM, IM, and PM. The rate of sodium excretion through urine at steady-state was high. This was consistent with the prediction that torsemide was metabolized mainly by CYP2C9 and would show different PK and PD patterns depending on different CYP2C9 phenotypes [5]. However, quantitative differences in PKs and PDs of torsemide at steady-state between CYP2C9 phenotypes were not very significant. That is, differences in the average torsemide concentration in plasma (or serum) between groups at steady-state were 1.17–1.20 times, and differences in the average urine sodium excretion rate at steady-state were 1.08–1.22 times, which were not large. This suggested that genetic polymorphisms of CYP2C9 did not have a very high degree of effect on PKs or PDs of torsemide. Past studies [5] have reported that genetic polymorphisms of CYP2C9 are effectively related to the PK-diversity of torsemide within a population. However, to what extent the genetic polymorphisms of CYP2C9 have an effect, particularly in terms of torsemide PDs, has not been reported. In this study, it was very interesting and important to be able to predict PKs and PDs upon multiple exposures to torsemide according to different CYP2C9 phenotypes and to identify differences between each phenotype. As a result, it was found that the proposed torsemide PBPK-PD model could be used to predict PKs and PDs of torsemide according to genetic polymorphisms of CYP2C9 and to elucidate differences when torsemide was administered once or repeatedly.

**Figure 4:**
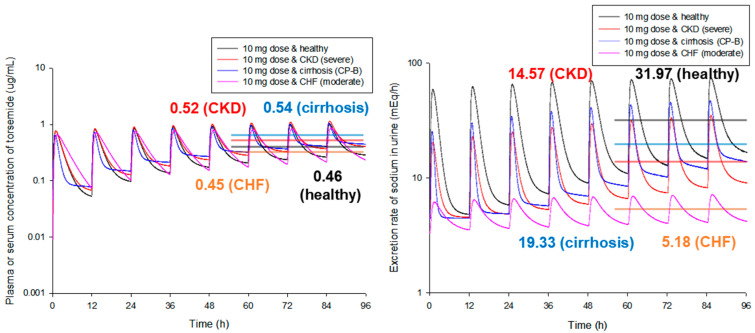
Simulation of plasma (or serum) torsemide concentration (**left**) and urinary sodium excretion rate (**right**) following multiple oral exposures (with 12 h dosing interval) of 10 mg torsemide in healthy adults, CKD patients, cirrhosis patients, and CHF patients. The line presented parallel to the X-axis means values in the steady-state.

Figure 4 presents simulation results of torsemide PKs and PDs between groups (healthy and patients) at steady-state according to the same exposure scenario (the same exposure dose of torsemide at 10 mg and the same exposure interval of 12 h). As a result of the model simulation comparison between healthy adult groups and various disease groups, there was no significant difference in plasma (or serum) torsemide concentration between groups (approximately 1.20-fold). However, there was a significant difference in the urinary sodium excretion rate (approximately 6.17-fold). This was probably because there were differences in PD-efficacy and potency of torsemide between groups. Here, PD-prediction for CKD patients was performed by applying the established PD-model to healthy adult populations. As a result of model simulation, it was confirmed that when torsemide was administered orally at the same dose and regimen, the sodium diuretic effect was low with the lowest torsemide plasma (or serum) concentration in the CHF patient groups. Considering the PD-aspect of torsemide, these results imply that the dose or frequency of administration of torsemide should be increased for CHF patients than for healthy adults. It was confirmed that the difference in torsemide PDs according to disease groups was more important than that according to CYP2C9 phenotypes. That is, torsemide PKs showed no significant differences by CYP2C9 phenotypes or disease groups. However, torsemide PDs showed significant differences by disease groups. These results suggest that considering disease groups is very important for setting the dosage and dosing of torsemide. In other words, the torsemide PBPK-PD model established in this study shows that when torsemide is used for cirrhosis, CKD, and CHF patients, it is not desirable to simply adjust the dosage and administration according to blood torsemide concentration monitoring results.

**Figure 5:**
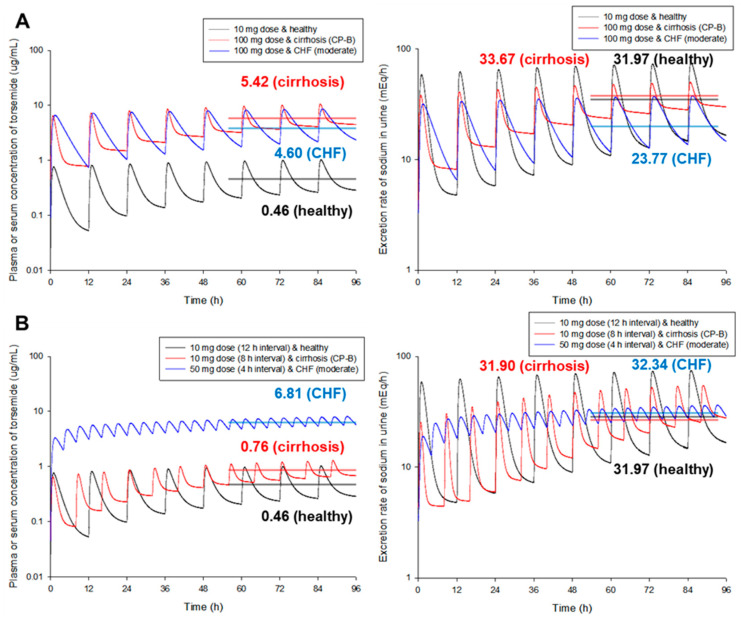
Simulation of plasma (or serum) torsemide concentration (left) and urinary sodium excretion rate (right) according to (**A**) administration dose and (**B**) frequency of torsemide multiple oral exposures in healthy adults, cirrhosis patients, and CHF patients. The line presented parallel to the X-axis means values in the steady-state.

Figure 5 shows PK and PD simulation results according to the dose and dosing interval of torsemide in healthy adults, cirrhosis patients, and CHF patients. Model simulation results confirmed that higher doses of torsemide were needed for cirrhosis and CHF patients to obtain an effect similar to that for healthy adult populations in terms of PDs. That is, when doses 10 times higher than that for healthy adult groups were administered to cirrhosis and CHF patient groups, average sodium urine excretion rates in the cirrhosis patient group (33.67 mEq/h) and CHF patient group (23.77 mEq/h) were similar to that of the healthy adult group (31.97 mEq/h). Although the difference between groups in PKs of torsemide was about 11.78 times (0.46 to 5.42), the difference in PDs was reduced to 1.42 times (33.67 to 23.77), which was a very interesting result. This suggests that in the case of torsemide, it is important to keep in mind that controlling the drug efficacy through blood level monitoring (PKs) is not clinically useful. It may lead to treatment failure. Instead, examining the pharmacological effect by measuring the amount of excreted urinary sodium or the volume of excreted urine could increase the clinical usefulness of torsemide and lead to successful treatment.

As a result of additional model simulations, it was confirmed that torsemide had to be administered more frequently or at higher doses in cirrhosis and CHF patient groups in order to obtain an effect similar to that of healthy adult groups in terms of PDs. That is, in the case of cirrhosis patient groups, when torsemide was administered at intervals of 8 h, it was confirmed that the average sodium urine excretion was similar to that of healthy adult groups administered at intervals of 12 h. In the case of CHF patient groups, it was confirmed that the average sodium urine excretion was similar to that of the healthy adult groups when the dose was five times higher (at 50 mg) than that of healthy adult groups with a frequency of 4 h. According to torsemide administration frequency or dose change, the difference in plasma (or serum) PKs between groups was 14.80 times (0.46 to 6.81). However, it was confirmed that the difference in PDs was reduced to 1.01 times (31.90 to 32.34). This was an important result, suggesting that differences in torsemide PKs in healthy adults and various patient groups did not reflect a proportional PD-effect. In other words, it implied that within the same categorical group, the PD-difference could be predicted in a proportional relationship only with PKs of torsemide. For different categorical groups (especially different patient groups), the PD-difference could not be easily predicted with PK-difference alone.

A limitation of this study was that the parameter changes applied to predict torsemide PKs and PDs in the patient groups did not fully reflect all actual factors. In reality, many physiological and biochemical factors could change with large individual differences depending on patient groups. However, it was not suitable for the scale of this model to consider all physiological factors. In this study, modeling and simulation of torsemide PKs and PDs were performed by partially reflecting well-known systemic factors for each patient group. In order to explain all physiological phenomena as models, further experimental studies of parameter-related information as well as bulk-up of the model size to explain detailed body actions will be needed. Although the bulk-up of the model and the addition of detailed parameters will increase the complexity of the model, it will more precisely predict PKs and PDs of torsemide. Nevertheless, the established PBPK-PD model for torsemide in this study was applicable not only to healthy adult populations but also to various patient populations. This is very important in that it is a very simple and efficient modeling approach that could be widely applied to many exposure scenarios or clinical cases. Another limitation of this study was the limitation of the data that could be accessed. In particular, for PD-data of torsemide, it will be necessary to conduct clinical studies of cirrhosis and CHF patients with different severities (such as a very severe group). The torsemide PD-model could be further improved through clinical investigation of other effective factors besides urinary sodium excretion as a PD-indicator of torsemide. In addition, this study has limitations in modeling and simulating torsemide PKs and PDs according to the application of torsemide alone. In actual clinical practice, combining with other drugs is frequently performed. Thus, a quantitative prediction study considering its interactions with various drugs will be needed in the future. Such studies will expand its clinical applicability.

## 4. Conclusions

In this study, a PBPK-PD model for torsemide was established based on several invitro and clinical data from healthy adults. By reflecting physiological and biochemical changes according to cirrhosis, CKD, and CHF patient groups in the model, PKs and PDs of torsemide for each patient group were further simulated. The PBPK-PD model established in this study captured well theresults of various torsemide clinical data overall. Simulation of torsemide PKs and PDs according to different CYP2C9 phenotypes in healthy adult populations confirmed that the effect of CYP2C9 genetic polymorphisms on torsemide PK and PD diversities was not as great as the difference between disease groups. The difference in torsemide PDs according to disease groups was larger than that of PK. The absence of PD proportional to the degree of PK-difference suggests that it is very important to consider disease groups during the clinical application of torsemide. According to the simulation results of the torsemide PBPK-PD model, it was necessary to increase the dose and frequency of torsemide administration for cirrhosis and CHF patients in order to show the same level of PDs as in healthy adults. The significance of this study was that it provided us an opportunity to predict PKs and PDs of torsemide for various groups and to establish quantitative clinical therapies based on modeling. The PBPK-PD model for torsemide established in this study can be expanded and applied to drug-drug interaction studies with other concomitant drugs.

## Figures and Tables

**Table 1:** Metabolic parameter values for each CYP2C9 phenotype reflected in the torsemide PBPK-PD model.

Description	Parameters	Value	Unit	Source
Maximum metabolic rate constant for EM	V_max, EM_	2.216	μg/mL/h	Estimated *
Michaelis-Menten constant for EM	K_m, EM_	1.951	μg/mL	Estimated *
Maximum metabolic rate constant for IM	V_max, IM_	0.886	μg/mL/h	Estimated *
Michaelis-Menten constant for IM	K_m, IM_	5.073	μg/mL	Estimated *
Maximum metabolic rate constant for PM	V_max, PM_	0.222	μg/mL/h	Estimated *
Michaelis-Menten constant for PM	K_m, PM_	8.242	μg/mL	Estimated *

* indicated estimated values based on the literature data [6,33] and model simulations (fitting to observations).

**Table 2:** Changes of physiological and biochemical parameters related to hepatic cirrhosis.

Parameters	Control	Child-Pugh (CP) Class		
		A	B	C
Liver volume fraction *	1	0.81	0.65	0.53
Cardiac output fraction *	1	1.16	1.32	1.41
Kidney blood flow rate fraction *	1	0.70	0.58	0.55
Portal blood flow rate fraction *	1	0.91	0.63	0.55
Hepatic arterial blood flow rate fraction *	1	1.41	1.62	1.91
Villous blood flow rate fraction *	1	1.29	1.52	1.99
Hematocrit (%) **	43	38.46	34.51	33.51
Albumin & α_1_-acid glycoprotein (g/L) **	50.34	46.33	38.18	29.69
CYP2C9 (pmol/mg) *	73	50.4	38.0	24.1

Control meant healthy populations not included in any cirrhosis classifications. Cirrhosis patient groups were classified according to the Child–Pugh (CP) classification system [42]. * denoted values derived from previous reports [37,38,39,40]. ** denoted values derived from populations embedded in the Simcyp^TM^ PBPK Simulator (version 21).

**Table 3:** Changes of physiological and biochemical parameters related to CKD patient groups.

Parameters	Control	CKD Class		
		Mild	Moderate	Severe
Kidney volume fraction *	1	0.99	0.93	0.87
Kidney blood flow rate fraction *	1	0.99	0.86	0.72
Liver intrinsic clearance fraction *	1	1.00	0.69	0.52
Renal elimination rate fraction *	1	0.99	0.68	0.37

Control meant healthy populations not included in any CKD classifications. CKD patient groups were classified according to their glomerular filtration rate (GFR) levels [48]. Patients with GFR values of 60–89, 30–59, and <30 mL/min/1.73 m^2^ were considered mild, moderate, and severe, respectively. * denoted values derived from previous reports [45,46,47].

**Table 4:** Changes of physiological and biochemical parameters related to CHF patient groups.

Parameters	Control	CHF Class		
		Mild	Moderate	Severe
Cardiac output fraction *	1	0.69	0.50	0.42
Kidney blood flow rate fraction *	1	0.78	0.55	0.63
Portal blood flow rate fraction *	1	0.76	0.54	0.46
Hepatic arterial blood flow rate fraction *	1	0.76	0.54	0.46
Other tissues blood flow rate fraction *	1	0.57	0.44	0.28
Liver intrinsic clearance fraction *	1	0.76	0.54	0.46

Control meant healthy populations not included in any CHF classifications. CHF patient groups were classified according to the New York Heart Association (NYHA) classification system [52]. NYHA classes II, III, and IV were considered mild, moderate, and severe, respectively. * denoted values derived from previous reports [50,51].

**Table 5:** Torsemide PD-model and parameter values in healthy adult populations, cirrhosis patients, and CHF patients.

Baseline Sigmoid E_max_ Model	Populations		
	Healthy *	Cirrhosis **	CHF ***
Equation	$E=E_{0}+\frac{E_{max}}{{\left(1+\frac{x}{EC_{50}}\right)}^{P}}$		
Parameters			
*E*_0_ (mEq/h)	4.39	4.33	3.25
*E_max_* (mEq/h)	105.1	65.24	83.45
*EC*_50_ (μg/h)	345.5	388.8	3985
*P*	−2.13	−2.82	−1.16

*E* means urinary excretion rate of sodium. *E*_0_ means basal urinary excretion rate of sodium. *E_max_* is the maximum urinary excretion rate of sodium. *EC*_50_ is the value of the urinary excretion rate of torsemide equal to 50% of the maximum urinary excretion rate of sodium. *x* means urinary excretion rate of torsemide. *P* means the slope constant of the graph. * indicates that the PD-model is established based on previously reported data [9,10,11,14] on urinary torsemide excretion rates and sodium excretion rates in healthy adults. ** indicates that the PD-model is established based on previously reported data [10] on urinary torsemide excretion rates and sodium excretion rates in cirrhosis patients (CP-B). *** indicates that the PD-model is established based on previously reported data [12,13,26] on urinary torsemide excretion rates and sodium excretion rates in CHF patients (mild-moderate).

## Data Availability

All data generated or analyzed during this study are included in this published article and its Supplementary Information.

## Supplementary Materials

The following supporting information can be downloaded at: https://www.mdpi.com/article/10.3390/pharmaceutics14122720/s1, Table S1: Information on datasets applied for torsemide PBPK-PD modeling. Table S2: Information on parameters constituting the PBPK-PD model of torsemide for healthy adults. Table S3: Basic physicochemical parameters of torsemide. Figure S1: Relationship graph of urinary sodium excretion rate according to urinary torsemide excretion rate in (**A**) healthy adult groups, (**B**) cirrhosis, and (**C**) CHF patient groups. R^2^ and AIC mean the model correlation coefficient and model fitting values, respectively. The dark pink and light pink regions mean 95% confidence intervals and 95% prediction intervals, respectively. Figure S2: K_p_ values of (**A**) major tissue and (**B**) other tissues in humans predicted using Simcyp^TM^ PBPK Simulator and comparison with previously reported values in rats. Figure S3: Plasma or serum concentration profile predicted by the PBPK model and observations following single oral (up) or intravenous (down) exposure to torsemide (5, 10, and 20 mg) in healthy adult groups. Multicolored dots and black solid line represent individual (or mean) observed values and the mean predicted by the model, respectively. Figure S4: Plasma or serum concentration profile predicted by the PBPK model and observations following multiple oral exposures to torsemide (10 mg) in a healthy adult group. Yellow dots and black solid line represent observed mean values and the mean predicted by the model, respectively. The red area on the graph represents a 95% confidence interval. Figure S5: Cumulative urinary excretion profile predicted by the PBPK model and observations following single oral or intravenous exposure to torsemide (10 and 20 mg) in healthy adult groups. Dots (black or red colored) and black solid line represent observed average values and the mean predicted by the model, respectively. Figure S6: Urinary sodium excretion rate profile predicted by the PBPK-PD model and observations following single (**A**) oral or (**B**) intravenous exposure to torsemide (5, 10, and 20 mg) in healthy adult groups. Black dots represent the observed mean values following oral or intravenous exposure to 10 mg torsemide. Black, red, and blue solid lines mean average values at 5, 10, and 20 mg doses predicted by the model, respectively. Figure S7: Comparison graph of dose-normalized AUC_0–∞_ according to phenotypic combinations of CYP2C9 and OATP1B1. Comparison between EM and IM in the (**A**) ET population; (**B**) Comparison between EM and IM in the IT population; (**C**) Comparison between ET and IT in the EM population; (**D**) Comparison between ET, IT, and PT in the IM population. *, *p* < 0.05 by Student’s *t*-test. Figure S8: Plasma or serum concentration profiles of torsemide according to CYP2C9 phenotypes [(**A**) EM, (**B**) IM, and (**C**) PM] in healthy adult populations (following single oral exposure to 5, 10, and 20 mg torsemide). Multicolored dots represent individual observed values or mean values. Black, red, and pink solid lines mean average values at 5, 10, and 20 mg doses predicted by the model, respectively. Figure S9: Prediction of (**A**) the cumulative urinary excretion amount and (**B**) the urinary sodium excretion rate of torsemide according to CYP2C9 phenotypes after oral administration of 10 mg torsemide. Here, PD prediction (as sodium excretion rate through urine) according to CYP2C9 phenotype was performed based on the PD model for healthy adults. Figure S10: Changes in model parameters by reflecting physiological and biochemical changes according to CP-A, CP-B, and CP-C in the cirrhosis patient group. Figure S11: Plasma or serum concentration (up) and cumulative urinary excretion (down) profiles of torsemide according to severity (as CP-A, CP-B, and CP-C) in cirrhosis patient groups and healthy adult group [following single (**A**) oral or (**B**) intravenous exposure to 10 mg torsemide]. Red dots represent observed mean values (in CP-B). Black, red, and blue solid lines mean average values in CP-A, CP-B, and CP-C cirrhosis predicted by the model, respectively. Pink solid line means average values in the healthy adult group predicted by the model. Figure S12: Urinary excretion rate profiles of sodium in CP-B cirrhosis patient group [following single (**A**) oral or (**B**) intravenous exposure to 10 mg torsemide]. Red dots and solid lines represent observed mean values and average values predicted by the model, respectively. Blue and pink dotted lines mean E_0_ values in cirrhotic patients and healthy adult groups, respectively. Figure S13: Prediction of urinary sodium excretion rate according to cirrhosis grade (as CP-A or CP-B or CP-C) after (**A**) oral or (**B**) intravenous administration of torsemide at 10 mg. Cyan and pink dotted lines mean E_0_ values in cirrhotic patients and healthy adult groups, respectively. Figure S14: Changes in model parameters by reflecting physiological and biochemical changes according to mild, moderate, and severe CKD. Figure S15: Plasma or serum concentration (up) and cumulative urinary excretion (down) profiles of torsemide according to severity (as mild, moderate, and severe) in CKD patient groups [following single (**A**) oral or (**B**) intravenous exposure to 100 mg torsemide]. Dots (red or blue colored) represent observed mean values. Black, red, and blue solid lines mean average values in mild, moderate, and severe CKD predicted by the model, respectively. Figure S16: Changes in model parameters by reflecting physiological and biochemical changes according to mild, moderate, and severe CHF. Figure S17: Plasma or serum concentration-time profiles of torsemide according to severity (as mild, moderate, and severe) in CHF patient groups [following single (**A**) oral or (**B**) intravenous exposure to 10 or 100 mg torsemide]. Dots (red or blue colored) represent observed mean values. Black, red, and blue solid lines mean average values in mild, moderate, and severe CHF predicted by the model, respectively. Figure S18: Cumulative urinary excretion-time profiles of torsemide according to severity (as mild, moderate, and severe) in CHF patient groups [following single (**A**) oral or (**B**) intravenous exposure to 10–200 mg torsemide]. Dots (red or blue colored) represent observed mean values. Black, red, and blue solid lines mean the average values in mild, moderate, and severe CHF predicted by the model, respectively. Figure S19: Urinary excretion rate profiles of sodium in CHF (**A**) moderate and (**B**,**C**) mild or severe patient groups [following single (**A**,**B**) oral exposure to 50–200 mg torsemide or (**C**) intravenous administration of 5–20 mg torsemide]. Multicolored dots and solid lines represent observed mean values and average values predicted by the model, respectively. Figure S20: Relationship graphs between torsemide PKs and PDs in (**A**) healthy adults, (**B**) cirrhosis (CP-B), and (**C**) CHF (moderate) patients following oral (left) or intravenous (right) administration of 10 mg torsemide (predicted using the established PBPK-PD model). Three-dimensional relationship (up) between torsemide plasma (or serum) concentration and urinary excretion rate and urinary sodium excretion rate at the same time point are shown. Two-dimensional relationships (medium and down) between torsemide plasma (or serum) concentration or urinary torsemide excretion rate and urinary sodium excretion rate at the same time point are shown. Dots and solid lines mean values predicted by torsemide’s PBPK-PD model and connecting those values, respectively. Figure S21: Sensitivity coefficients according to variation of each model parameter based on (**A**) AUC_0–∞_ and (**B**) C_max_ of torsemide at doses of 5, 10, 50, 100, and 200 mg.

## Appendix A

Differential mass balance equations were expressed for individual tissues. These equations showed the mass balance of torsemide in the blood pool, liver, kidney, GI-tract, lung, and rest of body compartments, oral compartment, and urinary excretion compartments after oral or intravenous administration of torsemide:

- *Blood pool compartment:*

(A1) $$\frac{dC_{bl}}{dt}=\left(\begin{array}{c} F_{r}\cdot P_{r}\cdot Q_{lu}\cdot \frac{C_{lu}}{K_{lu}}+F_{r}\cdot P_{r}\cdot Q_{ki}\cdot \frac{C_{ki}}{K_{ki}}+F_{r}\cdot P_{r}\cdot Q_{live}\cdot \frac{C_{li}}{K_{li}}\\ +F_{r}\cdot P_{r}\cdot Q_{rb}\cdot \frac{C_{rb}}{K_{rb}} \end{array}\right)/V_{bl}-\left(F_{r}\cdot P_{r}\cdot Q_{lu}\;+F_{r}\cdot P_{r}\cdot Q_{ki}+F_{r}\cdot P_{r}\cdot Q_{liar}+F_{r}\cdot P_{r}\cdot Q_{gi}+F_{r}\cdot P_{r}\cdot Q_{rb}\right)\cdot C_{bl}/V_{bl}$$

- *For intravenous injection:*

(A2)
$$C_{bl}\left(t=0\right)=dose/V_{bl}$$

- *For the oral compartment:*

(A3)
$$\frac{dA_{oral}}{dt}=-K_{a}\cdot A_{oral}$$

(A4)
$$A_{oral}\left(t=0\right)=dose$$

- *GI-tract compartment (oral dosing):*

(A5)
$$\frac{dC_{gi}}{dt}=(F_{r}\cdot P_{r}\cdot Q_{gi}\cdot C_{bl}-F_{r}\cdot P_{r}\cdot Q_{gi}\cdot \frac{C_{gi}}{K_{gi}}+K_{a}\cdot A_{oral})/V_{gi}-K_{e}\cdot C_{gi}$$

- *Liver compartment:*

(A6)
$$\frac{dC_{li}}{dt}=(F_{r}\cdot P_{r}\cdot Q_{liar}\cdot C_{bl}-F_{r}\cdot P_{r}\cdot Q_{live}\cdot \frac{C_{li}}{K_{li}}+F_{r}\cdot P_{r}\cdot Q_{gi}\cdot \frac{C_{gi}}{K_{gi}})/V_{li}-\frac{V_{max}\cdot C_{li}}{K_{m}+C_{li}}$$

- *Kidney compartment:*

(A7)
$$\frac{dC_{ki}}{dt}=\left(F_{r}\cdot P_{r}\cdot Q_{ki}\cdot C_{bl}-F_{r}\cdot P_{r}\cdot Q_{ki}\cdot \frac{C_{ki}}{K_{ki}}\right)/V_{ki}-K_{u}\cdot C_{ki}$$

- *Urinary excretion compartment:*

(A8)
$$\frac{dA_{u}}{dt}=K_{u}\cdot C_{ki}\cdot V_{ki}$$

- *Lung compartment:*

(A9)
$$\frac{dC_{lu}}{dt}=(F_{r}\cdot P_{r}\cdot Q_{lu}\cdot C_{bl}-F_{r}\cdot P_{r}\cdot Q_{lu}\cdot \frac{C_{lu}}{K_{lu}})/V_{lu}$$

- *Rest of body compartment:* including adipose, heart, spleen, muscle, etc.

(A10)
$$\frac{dC_{rb}}{dt}=(F_{r}\cdot P_{r}\cdot Q_{rb}\cdot C_{bl}-F_{r}\cdot P_{r}\cdot Q_{rb}\cdot \frac{C_{rb}}{K_{rb}})/V_{rb}$$

In these model equations, *A_oral_* is the amount of absorbed dose, *A_u_* is the cumulative urinary excretion amounts of torsemide, K_a_ is the oral absorption rate constant of torsemide, and the dose is the administered dose of torsemide. *V_bl_*, *V_ki_*, *V_li_*, *V_gi_*, *V_rb_*, and *V_lu_* are volumes of blood, kidneys, liver, GI-tract, rest of the body, and lungs, respectively. *C_bl_*, *C_ki_*, *C_li_*, *C_gi_*, *C_rb_*, and *C_lu_* are concentrations of torsemide in the blood, kidneys, liver, GI-tract, rest of the body, and lungs, respectively. *K_ki_*, *K_li_*, *K_gi_*, *K_rb_*, and *K_lu_* are *K_p_* values of torsemide in the kidneys, liver, GI-tract, rest of the body, and lungs, respectively, where *K_p_* means the partition coefficient of torsemide from blood to each tissue. *Q_bl_*, *Q_ki_*, *Q_gi_*, *Q_rb_*, and *Q_lu_* are blood flow rates in the blood, kidneys, GI-tract, the rest of the body, and lungs, respectively. *Q_live_* and *Q_liar_* are blood flow rates in the hepatic vein and artery, respectively. *K_e_* is the elimination rate constant of torsemide from GI-tract. *K_u_* is the urinary excretion rate constant of torsemide. *F_r_* is the unbound fraction of torsemide in plasma or serum. *P_r_* is the plasma-to-blood cells partition ratio of torsemide. *V_max_* is the maximum metabolic rate constant of torsemide in the liver. *K_m_* is the torsemide concentration at half *V_max_*.
