# Supplementary material for: Torsemide Pharmacometrics in Healthy Adult Populations Including CYP2C9 Genetic Polymorphisms and Various Patient Groups through Physiologically Based Pharmacokinetic-Pharmacodynamic Modeling
Source: Pharmaceutics. 2022 Dec 5;14(12):2720. doi: 10.3390/pharmaceutics14122720 (PMC9784843; doi:10.3390/pharmaceutics14122720)
Supplement: Supplementary file 1 [file pharmaceutics-14-02720-s001.zip › pharmaceutics-1984023-supplementary.pdf]

Torsemide Pharmacometrics in Healthy Adult Populations Including CYP2C9 Genetic Polymorphisms and Various Patient Groups Through Physiologically Based Pharmacokinetic-Pharmacodynamic Modeling

Seung-Hyun Jeong [1,†], Ji-Hun Jang [2,†] and Yong-Bok Lee [2,*]

[1] College of Pharmacy, Sunchon National University, 255 Jungang-ro, Suncheon-si, Jeollanam-do 57922, Republic of Korea
[2] College of Pharmacy, Chonnam National University, 77 Yongbong-ro, Buk-gu, Gwangju 61186, Republic of Korea
* Correspondence: leeyb@chonnam.ac.kr; Tel.: +82-62-530-2931
† These authors contributed equally to this work and are co-first authors.

Table S1. Information on datasets applied for torsemide PBPK-PD modeling.

**Healthy adult population**	**Reference**	**Description**	**Matrix**
	Jeong et al (2022)*	A total of 112 male adults (19-29 years)<br>Oral administration (5, 10, and 20 mg doses of torsemide)<br>CYP2C9 genetic polymorphism in torsemide's PK	Serum
	Vormfelde et al (2004) [1]	A total of 36 adults (19-68 years)<br>Oral administration (10 mg dose of torsemide)<br>CYP2C9 genetic polymorphism in torsemide's PK	Plasma
	Barr et al (1990) [2]	A total of 11 male adults (65-83 years)<br>Oral and intravenous administrations (5 mg dose of torsemide)	Plasma
	Kramer et al (1994) [3]	A total of 14 male adults (21-37 years)<br>Oral administrations (10 mg dose of torsemide)	Plasma and urine

Vargo et al (1995) [4]	A total of 16 adults (8 males and 8 females; 39-81 years)<br>Oral and intravenous administrations (10 mg dose of torsemide)	Plasma
Schwartz et al (1993) [5]	A total of 12 adults (11 males and 1 female; 38-66 years)<br>Oral and intravenous oral administrations (10 mg dose of torsemide)	Plasma and urine
Kang et al (2013) [6]	A total of 28 male adults (23.68 ± 1.79 years)<br>Oral administration (5 mg dose of torsemide)	Serum
Barbanoj et al (2009) [7]	A total of 16 adults (7 males and 9 females; 20-32 years)<br>Single and multiple (4 repeated dose) oral administrations (10 mg dose of torsemide)	Plasma
Shah et al (2017) [8]	A total of 10 adults (21-45 years)<br>Oral administrations (20 mg dose of torsemide)	Plasma and urine
Cho et al (2005) [9]	A total of 28 male adults (average 22.50 years)	Serum

		Oral administration (10 mg dose of torsemide)	
	Spahn et al (1990) [10]	A total of 7 adults (4 males and 3 females; 24-51 years)<br>Intravenous administrations (20 mg dose of torsemide)	Plasma
**CKD patient group**	**Reference**	**Description**	**Matrix**
	Gehr et al (1994) [11]	A total of 24 patients (18 males and 6 females; average 52.8-63.3 years)<br>Oral and intravenous administrations (100 mg dose of torsemide)	Plasma and urine
**Cirrhosis patient group**	**Reference**	**Description**	**Matrix**
	Schwartz et al (1993) [5]	A total of 12 patients (11 males and 1 female; 38-66 years)<br>Oral and intravenous oral administrations (10 mg dose of torsemide)	Plasma and urine
**CHF patient group**	**Reference**	**Description**	**Matrix**

	Vargo et al (1995) [4]	A total of 16 patients (8 males and 8 females; 39-81 years)<br>Oral and intravenous administrations (10 mg dose of torsemide)	Plasma and urine
	Vargo et al (1994) [12]	A total of 16 patients (11 males and 5 females; 41-78 years)<br>Oral administration (50, 100, and 200 mg doses of torsemide)	Urine
	Ballester et al (2015) [13]	A total of 10 patients (8 males and 2 females; 63.20 ± 12.44 years)<br>Oral administration (10 mg dose of torsemide)	Plasma
	Bleske et al (1998) [14]	A total of 12 patients<br>Oral administration (100 mg dose of torsemide)	Plasma and urine
	Kramer et al (1996) [15]	A total of 8 patients (7 males and 1 female; 44-65 years)<br>Intravenous administration (100 mg dose of torsemide)	Urine
	Hariman et al (1994) [16]	A total of 38 patients (32 males and 6 females; 27-82 years)<br>Intravenous administration (5, 10, and 20 mg doses of torsemide)	Urine

* means that individual data have been fully collected and applied.

Table S2. Information on parameters constituting the PBPK-PD model of torsemide for healthy adults.

Description	Parameter	Value	Unit	Source
Volume of blood	V_{bl}	5400	mL	Literature**
Volume of lung	V_{lu}	1200	mL	Literature**
Volume of kidney	V_{ki}	300	mL	Literature**
Volume of liver	V_{li}	1500	mL	Literature**
Volume of GI-tract	V_{gi}	2400	mL	Literature**
Volume of rest of body	V_{rb}	6.243×10^4	mL	Estimated***
Blood flow rate of blood vessel	Q_{bl}	3.798×10^5	mL/h	Literature**
Blood flow rate to lung	Q_{lu}	3.798×10^5	mL/h	Literature**
Blood flow rate to kidney	Q_{ki}	7.44×10^4	mL/h	Literature**
Blood flow rate to GI-tract	Q_{gi}	6.6×10^4	mL/h	Literature**
Blood flow rate to rest of body	Q_{rb}	1.524×10^5	mL/h	Estimated***

Blood flow rate to liver vein	Q_{live}	1.53×10^5	mL/h	Literature**
Blood flow rate to liver artery	Q_{liar}	8.7×10^4	mL/h	Literature**
Partition coefficient to lung	K_{lu}	0.23	-	Simulated*
Partition coefficient to kidney	K_{ki}	0.15	-	Simulated*
Partition coefficient to liver	K_{li}	0.11	-	Simulated*
Partition coefficient to GI-tract	K_{gi}	0.18	-	Simulated*
Partition coefficient to rest of body	K_{rb}	1.03	-	Simulated*
Unbound fraction in plasma or serum	F_r	0.05	-	Literature**
Plasma-to-blood cells partition ratio	P_r	0.28	-	Literature**
Maximum metabolic rate constant	V_{max}	1.11	µg/mL/h	Literature**
Michaelis-Menten constant	K_m	3.90	µg/mL	Literature**
Oral absorption rate constant	K_a	5.50	1/h	Estimated***
Elimination rate constant to urine	K_u	18.50	1/h	Estimated***

Elimination rate constant in GI-tract	K_e	1.00	1/h	Estimated***

* indicates values predicted using Simcyp^{TM} PBPK Simulator.

** denotes values derived from references [17-22].

*** means values estimated through fitting between observations and model simulation results.

Table S3. Basic physicochemical parameters of torsemide.

Parameter	Value	Source
Molecular weight (g/mol)	348.4	Literature*
Log P	0.57	Literature*
pKa	7.10	Literature*

* denotes values derived from references [23,24].

References

1. Vormfelde, S.V.; Engelhardt, S.; Zirk, A.; Meineke, I.; Tuchen, F.; Kirchheiner, J.; Brockmöller, J. CYP2C9 polymorphisms and the interindividual variability in pharmacokinetics and pharmacodynamics of the loop diuretic drug torsemide. *Clin. Pharmacol. Ther.* **2004**, *76*, 557-566.
2. Barr, W.; Smith, H.; Karnes, H.; Sica, D.; Vetticaden, S.; Purich, E.; Prasad, V.; Schary, W.; Kramer, W.; Linberg, S. Comparison of bioavailability, pharmacokinetics and pharmacodynamics of torasemide in young and elderly healthy volunteers. *Prog. Pharmacol. Clin. Pharmacol.* **1990**, *8*, 15-28.
3. Kramer, W. Pharmacokinetics and pharmacodynamics of torasemide in congestive heart failure. *Cardiology* **1994**, *84*, 108-114.
4. Vargo, D.L.; Kramer, W.G.; Black, P.K.; Smith, W.B.; Serpas, T.; Brater, D.C. Bioavailability, pharmacokinetics, and pharmacodynamics of torsemide and furosemide in patients with congestive heart failure. *Clin. Pharmacol. Ther.* **1995**, *57*, 601-609.
5. Schwartz, S.; Brater, D.C.; Pound, D.; Green, P.K.; Kramer, W.G.; Rudy, D. Bioavailability, pharmacokinetics, and pharmacodynamics of torsemide in patients with cirrhosis. *Clin. Pharmacol. Ther.* **1993**, *54*, 90-97.
6. Kang, H.-A.; Yoon, H.; Lee, Y.-B. Bioequivalence of Torad tablet 5 mg to Torem tablet 5 mg (torasemide 5 mg). *J. Pharm. Investig.* **2013**, *43*, 153-159.
7. Barbanoj, M.; Ballester, M.; Antonijoan, R.; Gich, I.; Pelagio, P.; Gropper, S.; Santos, B.; Guglietta, A. Comparison of repeated-dose pharmacokinetics of prolonged-release and immediate-release torasemide formulations in healthy young volunteers. *Fundam. Clin. Pharmacol.* **2009**, *23*, 115-125.
8. Shah, S.; Pitt, B.; Brater, D.C.; Feig, P.U.; Shen, W.; Khwaja, F.S.; Wilcox, C.S. Sodium and fluid excretion with torsemide in healthy subjects is limited by the short duration of diuretic action. *J. Am. Heart. Assoc.* **2017**, *6*, e006135.
9. Cho, H.-Y.; Kang, H.-A.; Park, C.-H.; Kim, S.-M.; Kim, D.-H.; Park, S.; Kim, K.-R.; Hur, H.; Lee, Y.-B. Bioequivalence of Boryung torsemide tablet to Torem tablet (torasemide 10 mg) by high performance liquid chromatography/UV detector. *J. Pharm. Investig.* **2005**, *35*, 323-328.

10. Spahn, H.; Knauf, H.; Mutschler, E. Pharmacokinetics of torasemide and its metabolites in healthy controls and in chronic renal failure. *Eur. J. Clin. Pharmacol.* **1990**, *39*, 345-348.
11. Gehr, T.W.; Rudy, D.W.; Matzke, G.R.; Kramer, W.G.; Sica, D.A.; Brater, D.C. The pharmacokinetics of intravenous and oral torsemide in patients with chronic renal insufficiency. *Clin. Pharmacol. Ther.* **1994**, *56*, 31-38.
12. Vargo, D.; Kramer, W.G.; Black, P.K.; Smith, W.B.; Serpas, T.; Brater, D.C. The pharmacodynamics of torsemide in patients with congestive heart failure. *Clin. Pharmacol. Ther.* **1994**, *56*, 48-54.
13. Ballester, M.R.; Roig, E.; Gich, I.; Puntes, M.; Delgadillo, J.; Santos, B.; Antonijoan, R.M. Randomized, open-label, blinded-endpoint, crossover, single-dose study to compare the pharmacodynamics of torasemide-PR 10 mg, torasemide-IR 10 mg, and furosemide-IR 40 mg, in patients with chronic heart failure. *Drug Des. Devel. Ther.* **2015**, *9*, 4291-4302.
14. Bleske, B.E.; Welage, L.S.; Kramer, W.G.; Nicklas, J.M. Pharmacokinetics of torsemide in patients with decompensated and compensated congestive heart failure. *J. Clin. Pharmacol.* **1998**, *38*, 708-714.
15. Kramer, W.G.; Smith, W.B.; Ferguson, J.; Serpas, T.; Grant III, A.G.; Black, P.K.; Brater, D.C. Pharmacodynamics of torsemide administered as an intravenous injection and as a continuous infusion to patients with congestive heart failure. *J. Clin. Pharmacol.* **1996**, *36*, 265-270.
16. Hariman, R.J.; Bremner, S.; Louie, E.K.; Rogers, W.J.; Kostis, J.B.; Nocero, M.A.; Jones, J.P. Dose-response study of intravenous torsemide in congestive heart failure. *Am. Heart J.* **1994**, *128*, 352-357.
17. Davies, B.; Morris, T. Physiological parameters in laboratory animals and humans. *Pharm. Res.* **1993**, *10*, 1093-1095.
18. Igari, Y.; Sugiyama, Y.; Sawada, Y.; Iga, T.; Hanano, M. Prediction of diazepam disposition in the rat and man by a physiologically based pharmacokinetic model. *J. Pharmacokinet. Biopharm.* **1983**, *11*, 577-593.
19. Jeong, S.-H.; Jang, J.-H.; Cho, H.-Y.; Lee, Y.-B. Risk assessment for humans using physiologically based pharmacokinetic model of diethyl phthalate and its major

metabolite, monoethyl phthalate. *Arch. Toxicol.* **2020**, *94*, 2377-2400.
20. Jeong, S.-H.; Jang, J.-H.; Cho, H.-Y.; Lee, Y.-B. Human risk assessment of di-isobutyl phthalate through the application of a developed physiologically based pharmacokinetic model of di-isobutyl phthalate and its major metabolite mono-isobutyl phthalate. *Arch. Toxicol.* **2021**, *95*, 2385-2402.
21. Lee, D.Y.; Kim, J.Y.; Kim, Y.C.; Kwon, J.W.; Kim, W.B.; Lee, M.G. Dose-independent pharmacokinetics of torasemide after intravenous and oral administration to rats. *Biopharm. Drug Dispos.* **2005**, *26*, 173-182.
22. Miners, J.O.; Rees, D.; Valente, L.; Veronese, M.E.; Birkett, D.J. Human hepatic cytochrome P450 2C9 catalyzes the rate-limiting pathway of torsemide metabolism. *J. Pharmacol. Exp. Ther.* **1995**, *272*, 1076-1081.
23. Knauf, H.; Mutschler, E. Clinical pharmacokinetics and pharmacodynamics of torasemide. *Clin. Pharmacokinet.* **1998**, *34*, 1-24.
24. Thiel, C.; Schneckener, S.; Krauss, M.; Ghallab, A.; Hofmann, U.; Kanacher, T.; Zellmer, S.; Gebhardt, R.; Hengstler, J.G.; Kuepfer, L. A systematic evaluation of the use of physiologically based pharmacokinetic modeling for cross-species extrapolation. *J. Pharm. Sci.* **2015**, *104*, 191-206.

Supplementary Figure captions

Figure S1. Relationship graph of urinary sodium excretion rate according to urinary torsemide excretion rate in (A) healthy adult groups, (B) cirrhosis, and (C) CHF patient groups. R^2 and AIC mean the model correlation coefficient and model fitting values, respectively. The dark pink and light pink regions mean 95% confidence intervals and 95% prediction intervals, respectively.

Figure S2. K_p values of (A) major tissue and (B) other tissues in humans predicted using Simcyp^{TM} PBPK Simulator and comparison with previously reported values in rats.

Figure S3. Plasma or serum concentration profile predicted by the PBPK model and observations following single oral (up) or intravenous (down) exposure to torsemide (5, 10, and 20 mg) in healthy adult groups. Multicolored dots and black solid line represent individual (or mean) observed values and the mean predicted by the model, respectively.

Figure S4. Plasma or serum concentration profile predicted by the PBPK model and observations following multiple oral exposures to torsemide (10 mg) in healthy adult group. Yellow dots and black solid line represent observed mean values and the mean predicted by the model, respectively. The red area on the graph represents 95% confidence interval.

Figure S5. Cumulative urinary excretion profile predicted by the PBPK model and observations following single oral or intravenous exposure to torsemide (10 and 20 mg) in healthy adult groups. Dots (black or red colored) and black solid line represent observed average values and the mean predicted by the model, respectively.

Figure S6. Urinary sodium excretion rate profile predicted by the PBPK-PD model and observations following single (A) oral or (B) intravenous exposure to torsemide (5, 10, and 20 mg) in healthy adult groups. Black dots represent the observed mean values following oral or intravenous exposure of 10 mg torsemide. Black, red, and blue solid lines mean average values at 5, 10, and 20 mg doses predicted by the model, respectively.

Figure S7. Comparison graph of dose normalized $AUC_{0-\infty}$ according to phenotypic combinations of CYP2C9 and OATP1B1. Comparison between EM and IM in the (A) ET population; (B)

Comparison between EM and IM in the IT population; (C) Comparison between ET and IT in the EM population; (D) Comparison between ET, IT, and PT in the IM population. *, $p < 0.05$ by Student's *t*-test.

Figure S8. Plasma or serum concentration profiles of torsemide according to CYP2C9 phenotypes [(A) EM, (B) IM, and (C) PM] in healthy adult populations (following single oral exposure to 5, 10, and 20 mg torsemide). Multicolored dots represent individual observed values or mean values. Black, red, and pink solid lines mean average values at 5, 10, and 20 mg doses predicted by the model, respectively.

Figure S9. Prediction of (A) the cumulative urinary excretion amount and (B) urinary sodium excretion rate of torsemide according to CYP2C9 phenotypes after oral administration of 10 mg torsemide. Here, PD prediction (as sodium excretion rate through urine) according to CYP2C9 phenotype was performed based on the PD model for healthy adults.

Figure S10. Changes in model parameters by reflecting physiological and biochemical changes according to CP-A, CP-B, and CP-C in the cirrhosis patient group.

Figure S11. Plasma or serum concentration (up) and cumulative urinary excretion (down) profiles of torsemide according to severity (as CP-A, CP-B, and CP-C) in cirrhosis patient groups and healthy adult group [following single (A) oral or (B) intravenous exposure to 10 mg torsemide]. Red dots represent observed mean values (in CP-B). Black, red, and blue solid lines mean average values in CP-A, CP-B, and CP-C cirrhosis predicted by the model, respectively. Pink solid line means average values in healthy adult group predicted by the model.

Figure S12. Urinary excretion rate profiles of sodium in CP-B cirrhosis patient group [following single (A) oral or (B) intravenous exposure to 10 mg torsemide]. Red dots and solid lines represent observed mean values and average values predicted by the model, respectively. Blue and pink dotted lines mean E_0 values in cirrhotic patients and healthy adult groups, respectively.

Figure S13. Prediction of urinary sodium excretion rate according to cirrhosis grade (as CP-A or CP-B or CP-C) after (A) oral or (B) intravenous administration of torsemide at 10 mg. Cyan and

pink dotted lines mean E_0 values in cirrhotic patients and healthy adult groups, respectively.

Figure S14. Changes in model parameters by reflecting physiological and biochemical changes according to mild, moderate, and severe CKD.

Figure S15. Plasma or serum concentration (up) and cumulative urinary excretion (down) profiles of torsemide according to severity (as mild, moderate, and severe) in CKD patient groups [following single (A) oral or (B) intravenous exposure to 100 mg torsemide]. Dots (red or blue colored) represent observed mean values. Black, red, and blue solid lines mean average values in mild, moderate, and severe CKD predicted by the model, respectively.

Figure S16. Changes in model parameters by reflecting physiological and biochemical changes according to mild, moderate, and severe CHF.

Figure S17. Plasma or serum concentration-time profiles of torsemide according to severity (as mild, moderate, and severe) in CHF patient groups [following single (A) oral or (B) intravenous exposure to 10 or 100 mg torsemide]. Dots (red or blue colored) represent observed mean values. Black, red, and blue solid lines mean average values in mild, moderate, and severe CHF predicted by the model, respectively.

Figure S18. Cumulative urinary excretion-time profiles of torsemide according to severity (as mild, moderate, and severe) in CHF patient groups [following single (A) oral or (B) intravenous exposure to 10-200 mg torsemide]. Dots (red or blue colored) represent observed mean values. Black, red, and blue solid lines mean the average values in mild, moderate, and severe CHF predicted by the model, respectively.

Figure S19. Urinary excretion rate profiles of sodium in CHF (A) moderate and (B and C) mild or severe patient groups [following single (A and B) oral exposure to 50-200 mg torsemide or (C) intravenous administration of 5-20 mg torsemide]. Multicolored dots and solid lines represent observed mean values and average values predicted by the model, respectively.

Figure S20. Relationship graphs between torsemide PKs and PDs in (A) healthy adults, (B) cirrhosis (CP-B), and (C) CHF (moderate) patients following oral (left) or intravenous (right)

administration of 10 mg torsemide (predicted using the established PBPK-PD model). Three-dimensional relationship (up) between torsemide plasma (or serum) concentration and urinary excretion rate and urinary sodium excretion rate at the same time point are shown. Two-dimensional relationships (medium and down) between torsemide plasma (or serum) concentration or urinary torsemide excretion rate and urinary sodium excretion rate at the same time point are shown. Dots and solid lines mean values predicted by torsemide's PBPK-PD model and connecting those values, respectively.

Figure S21. Sensitivity coefficients according to variation of each model parameter based on (A) $AUC_{0-\infty}$ and (B) C_{max} of torsemide at doses of 5, 10, 50, 100, and 200 mg.

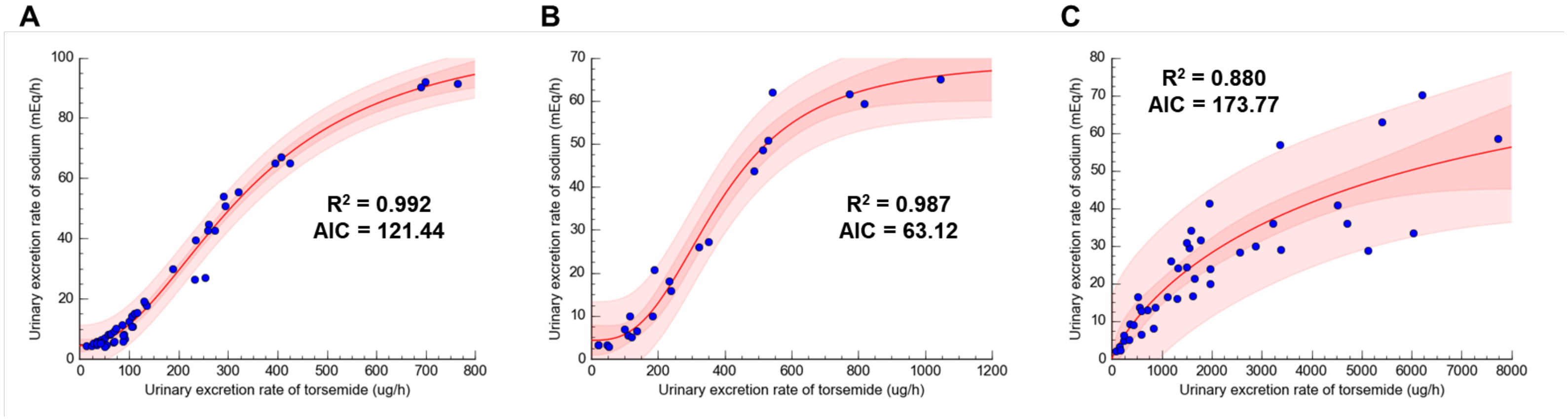

A
B
C
R^2 = 0.992
AIC = 121.44
R^2 = 0.987
AIC = 63.12
R^2 = 0.880
AIC = 173.77
Urinary excretion rate of sodium (mEq/h)
Urinary excretion rate of torsemide (ug/h)

Figure S1

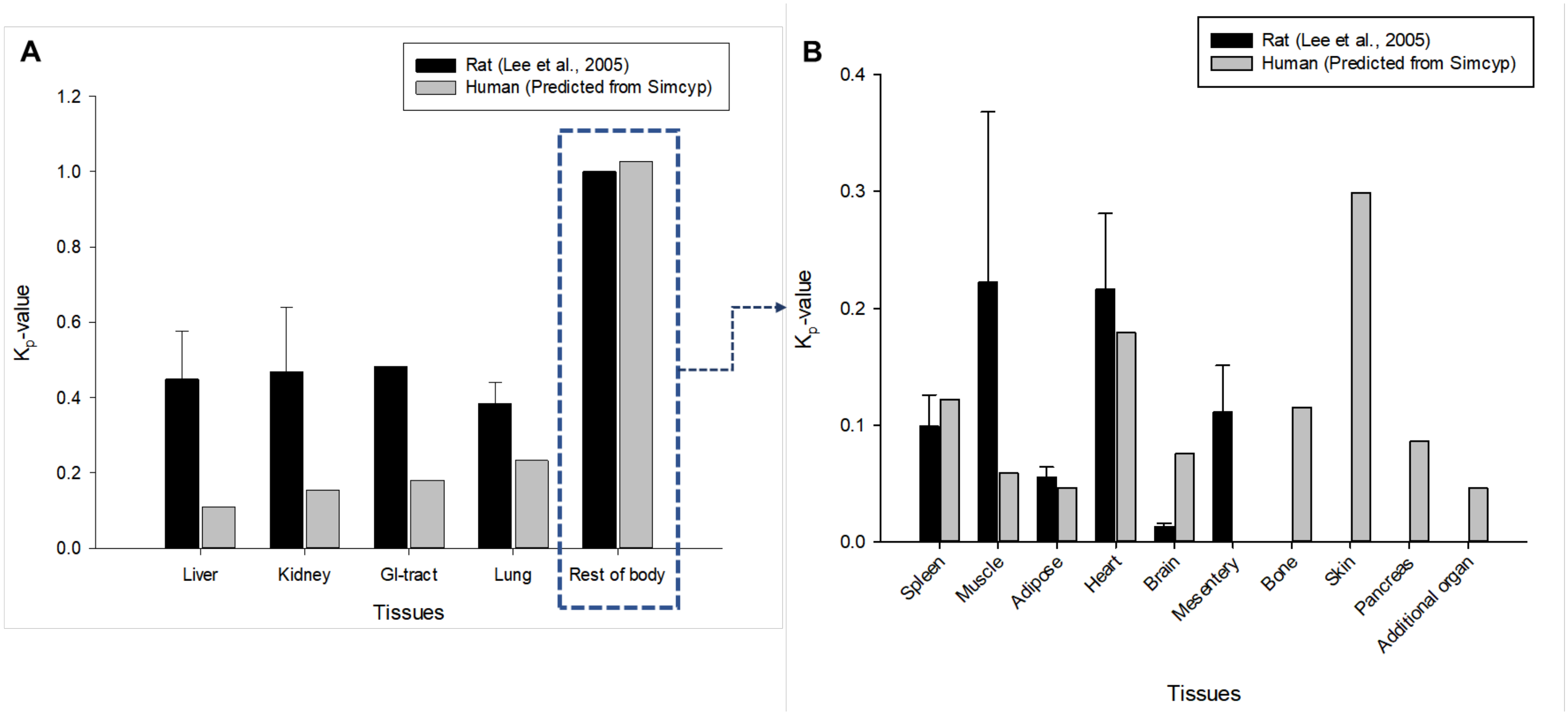
A
Rat (Lee et al., 2005)
Human (Predicted from Simcyp)
1.2
1.0
0.8
0.6
0.4
0.2
0.0
Kp-value
Liver
Kidney
GI-tract
Lung
Rest of body
Tissues
B
Rat (Lee et al., 2005)
Human (Predicted from Simcyp)
0.4
0.3
0.2
0.1
0.0
Kp-value
Spleen
Muscle
Adipose
Heart
Brain
Mesentery
Bone
Skin
Pancreas
Additional organ
Tissues

Figure S2

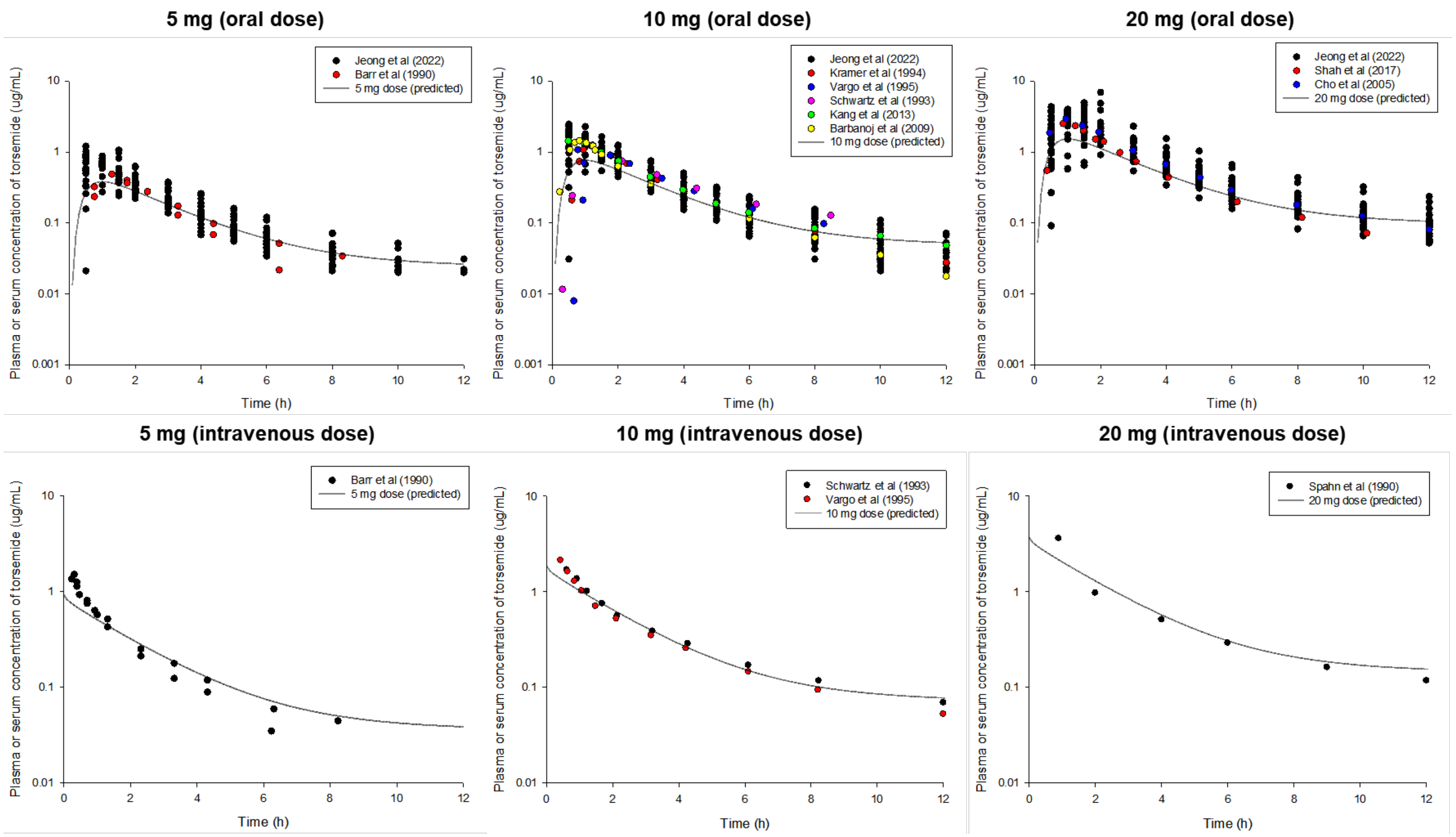
5 mg (oral dose)
Jeong et al (2022)
Barr et al (1990)
5 mg dose (predicted)
10 mg (oral dose)
Jeong et al (2022)
Kramer et al (1994)
Vargo et al (1995)
Schwartz et al (1993)
Kang et al (2013)
Barbanoj et al (2009)
10 mg dose (predicted)
20 mg (oral dose)
Jeong et al (2022)
Shah et al (2017)
Cho et al (2005)
20 mg dose (predicted)
5 mg (intravenous dose)
Barr et al (1990)
5 mg dose (predicted)
10 mg (intravenous dose)
Schwartz et al (1993)
Vargo et al (1995)
10 mg dose (predicted)
20 mg (intravenous dose)
Spahn et al (1990)
20 mg dose (predicted)
Plasma or serum concentration of torsemide (ug/mL)
Time (h)

Figure S3

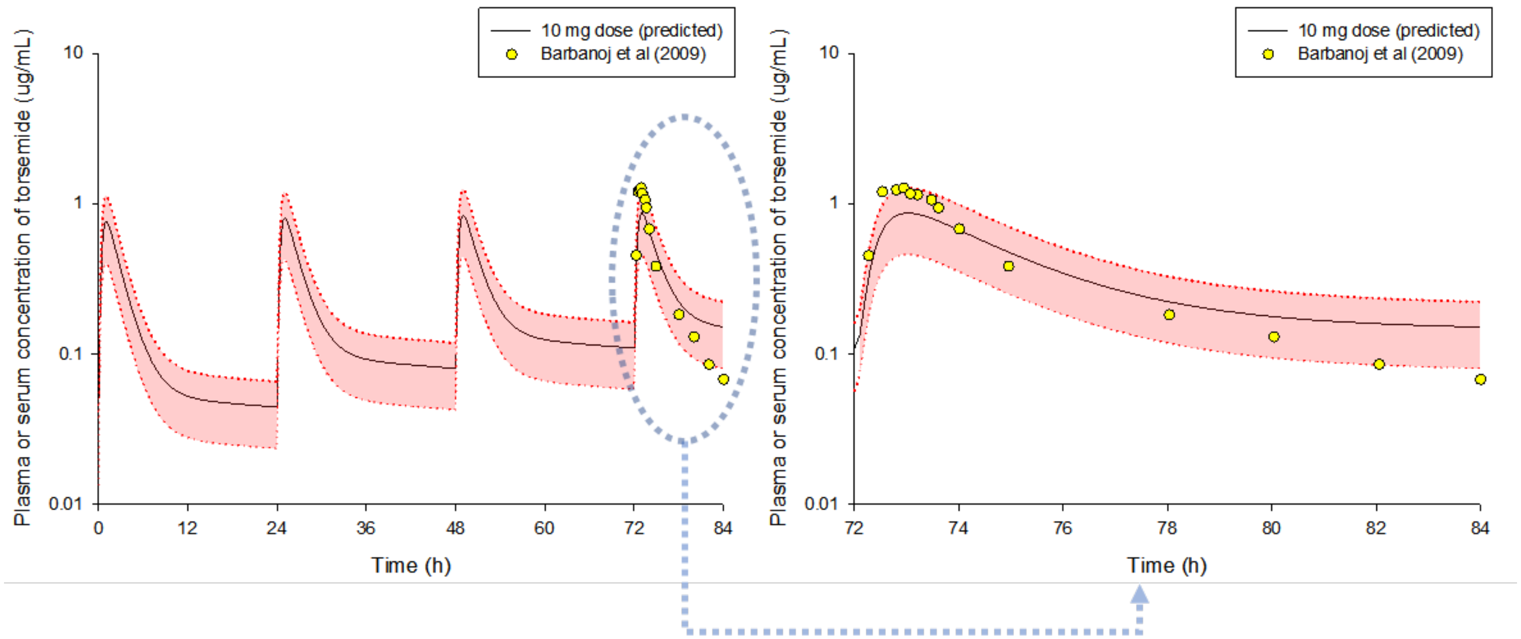
10 mg dose (predicted)
Barbanoj et al (2009)
Plasma or serum concentration of torsemide (ug/mL)
Time (h)
10
1
0.1
0.01
0
12
24
36
48
60
72
84
10 mg dose (predicted)
Barbanoj et al (2009)
Plasma or serum concentration of torsemide (ug/mL)
Time (h)
72
74
76
78
80
82
84

Figure S4

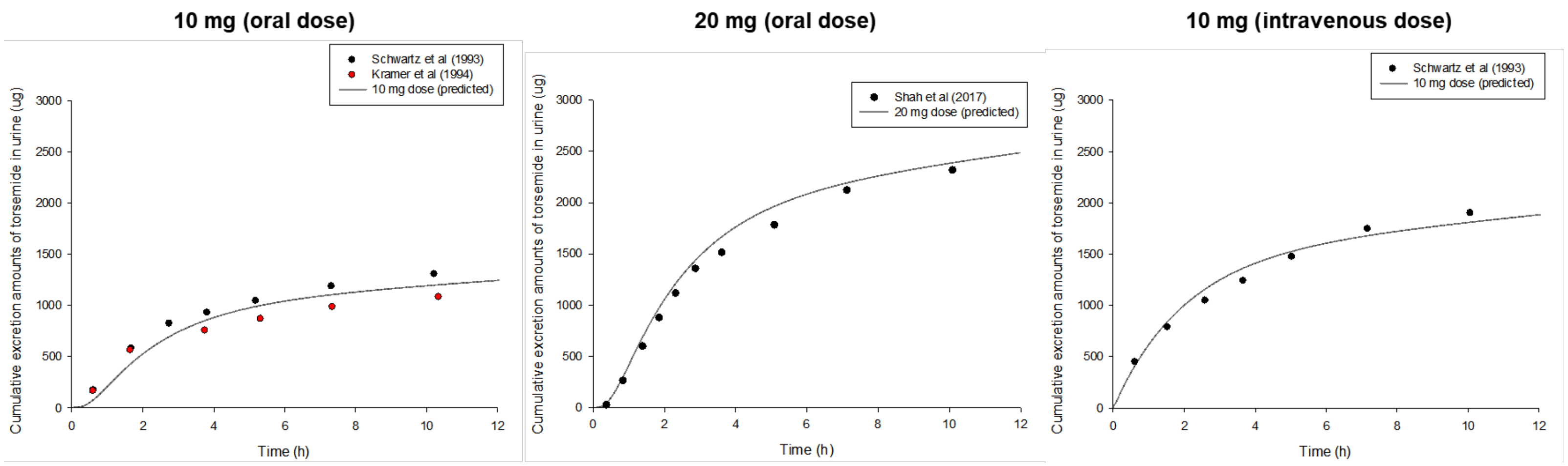
10 mg (oral dose)
Schwartz et al (1993)
Kramer et al (1994)
10 mg dose (predicted)
Cumulative excretion amounts of torsemide in urine (ug)
Time (h)
20 mg (oral dose)
Shah et al (2017)
20 mg dose (predicted)
Cumulative excretion amounts of torsemide in urine (ug)
Time (h)
10 mg (intravenous dose)
Schwartz et al (1993)
10 mg dose (predicted)
Cumulative excretion amounts of torsemide in urine (ug)
Time (h)

Figure S5

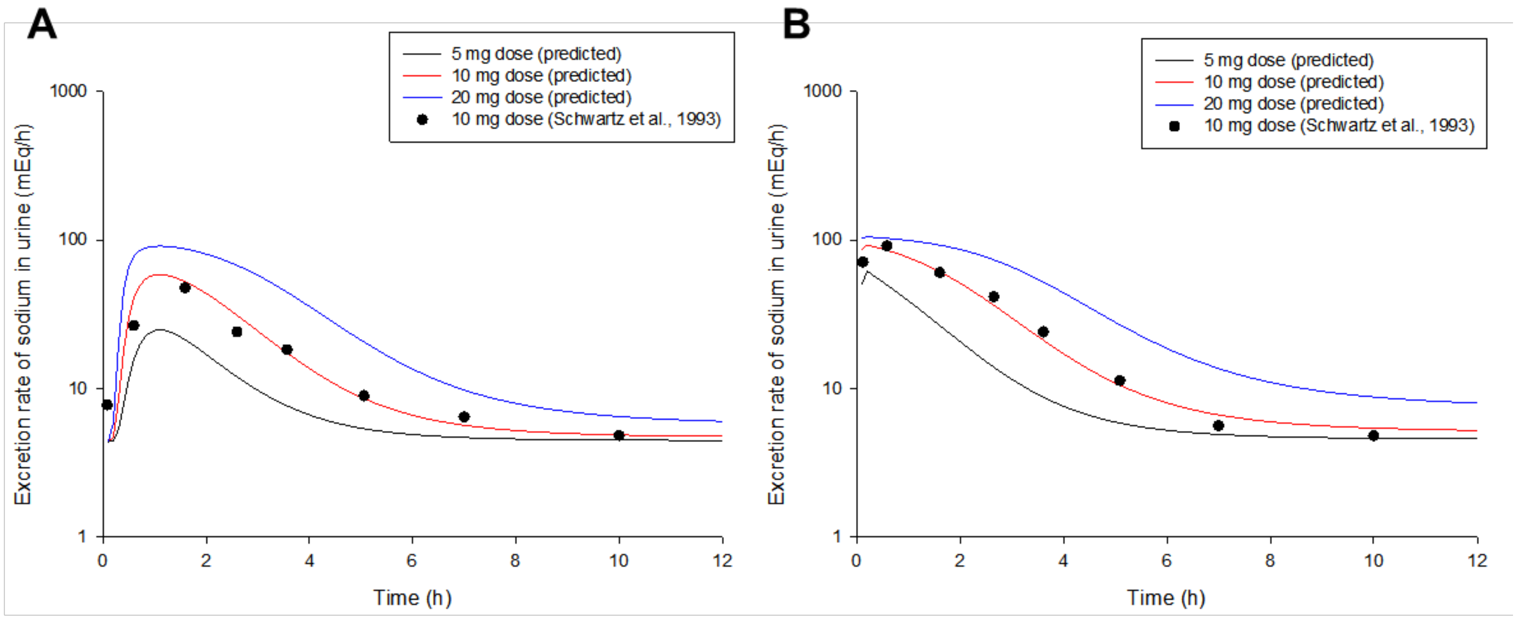
A
5 mg dose (predicted)
10 mg dose (predicted)
20 mg dose (predicted)
10 mg dose (Schwartz et al., 1993)
Excretion rate of sodium in urine (mEq/h)
1000
100
10
1
0
2
4
6
8
10
12
Time (h)
B
5 mg dose (predicted)
10 mg dose (predicted)
20 mg dose (predicted)
10 mg dose (Schwartz et al., 1993)
Excretion rate of sodium in urine (mEq/h)
Time (h)

Figure S6

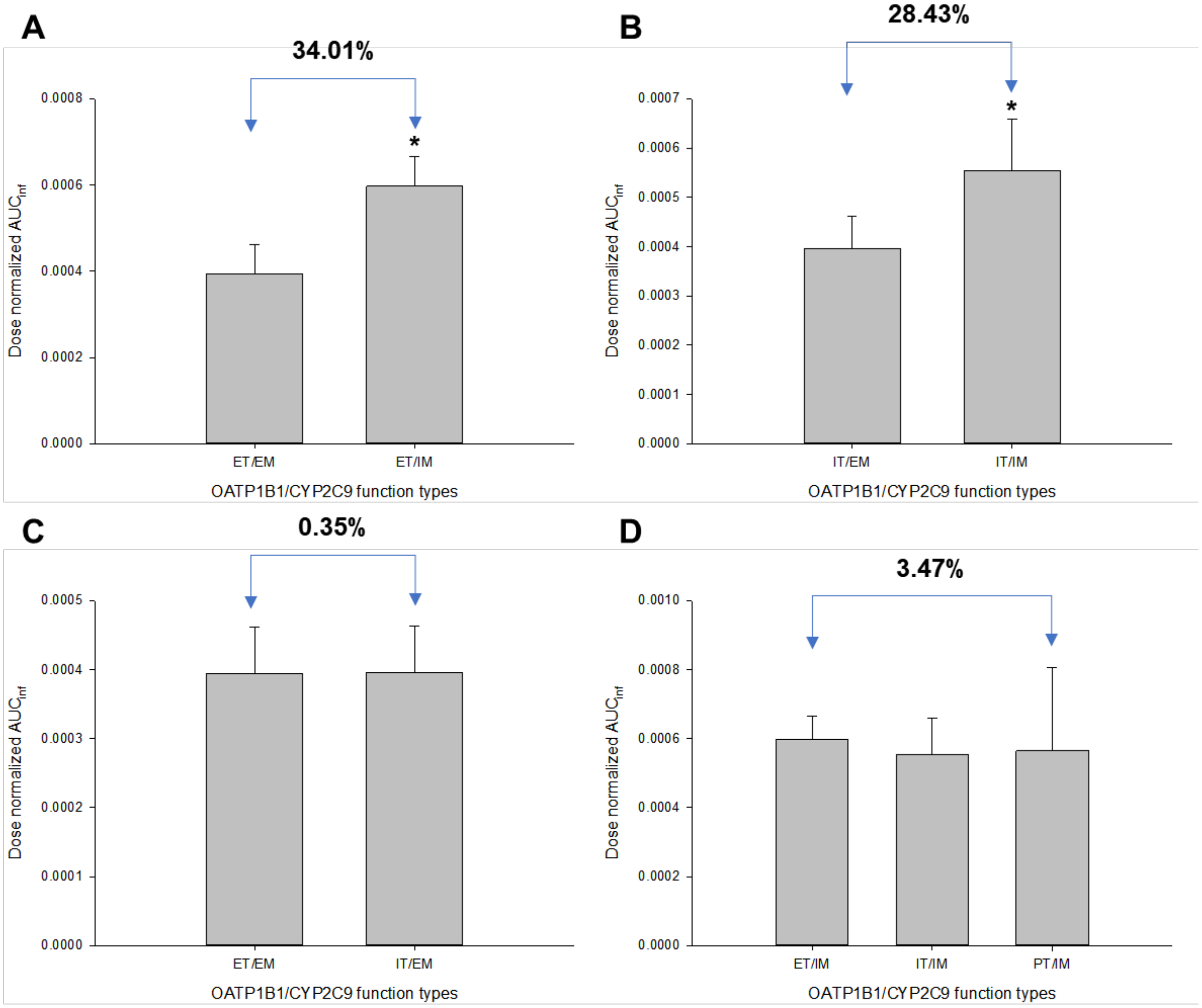
A
34.01%
0.0008
0.0006
0.0004
0.0002
0.0000
Dose normalized AUCinf
*
ET/EM
ET/IM
OATP1B1/CYP2C9 function types
B
28.43%
0.0007
0.0006
0.0005
0.0004
0.0003
0.0002
0.0001
0.0000
Dose normalized AUCinf
*
IT/EM
IT/IM
OATP1B1/CYP2C9 function types
C
0.35%
0.0005
0.0004
0.0003
0.0002
0.0001
0.0000
Dose normalized AUCinf
ET/EM
IT/EM
OATP1B1/CYP2C9 function types
D
3.47%
0.0010
0.0008
0.0006
0.0004
0.0002
0.0000
Dose normalized AUCinf
ET/IM
IT/IM
PT/IM
OATP1B1/CYP2C9 function types

Figure S7

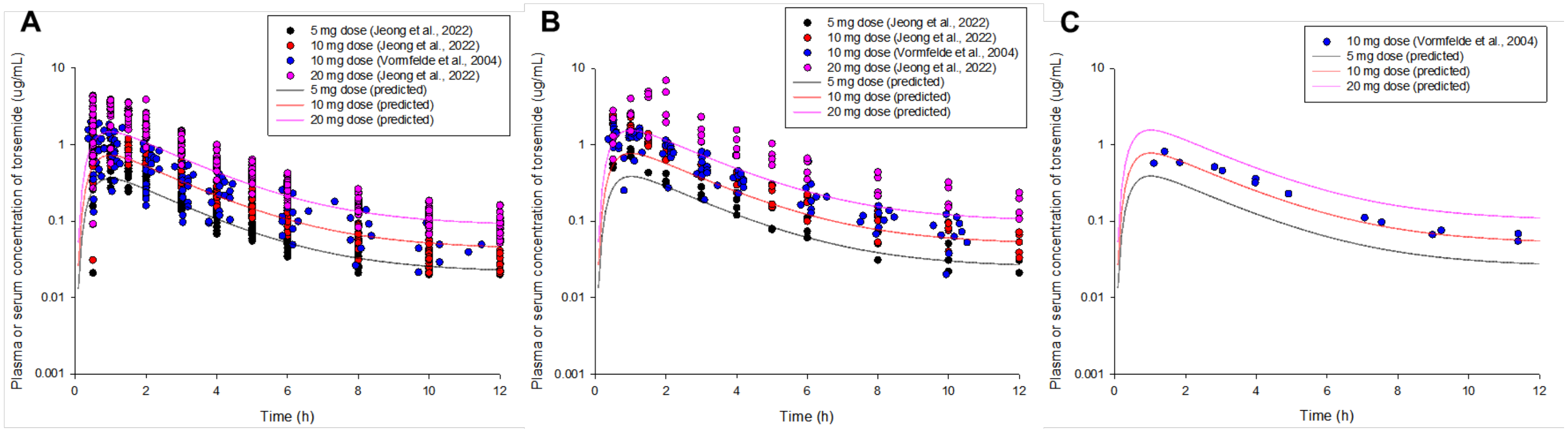

A
5 mg dose (Jeong et al., 2022)
10 mg dose (Jeong et al., 2022)
10 mg dose (Vormfelde et al., 2004)
20 mg dose (Jeong et al., 2022)
5 mg dose (predicted)
10 mg dose (predicted)
20 mg dose (predicted)
Plasma or serum concentration of torsemide (ug/mL)
Time (h)
B
5 mg dose (Jeong et al., 2022)
10 mg dose (Jeong et al., 2022)
10 mg dose (Vormfelde et al., 2004)
20 mg dose (Jeong et al., 2022)
5 mg dose (predicted)
10 mg dose (predicted)
20 mg dose (predicted)
Plasma or serum concentration of torsemide (ug/mL)
Time (h)
C
10 mg dose (Vormfelde et al., 2004)
5 mg dose (predicted)
10 mg dose (predicted)
20 mg dose (predicted)
Plasma or serum concentration of torsemide (ug/mL)
Time (h)

Figure S8

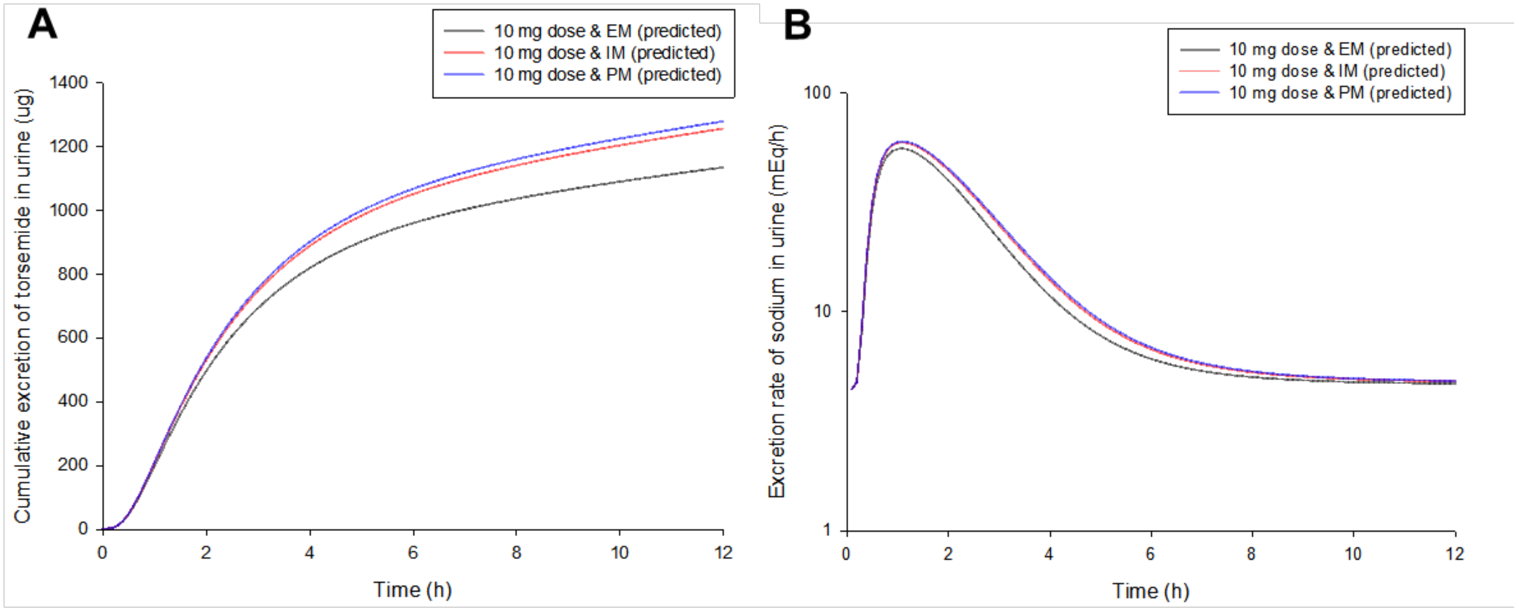

A
10 mg dose & EM (predicted)
10 mg dose & IM (predicted)
10 mg dose & PM (predicted)
Cumulative excretion of torsemide in urine (ug)
1400
1200
1000
800
600
400
200
0
0
2
4
6
8
10
12
Time (h)
B
10 mg dose & EM (predicted)
10 mg dose & IM (predicted)
10 mg dose & PM (predicted)
Excretion rate of sodium in urine (mEq/h)
100
10
1
0
2
4
6
8
10
12
Time (h)

Figure S9

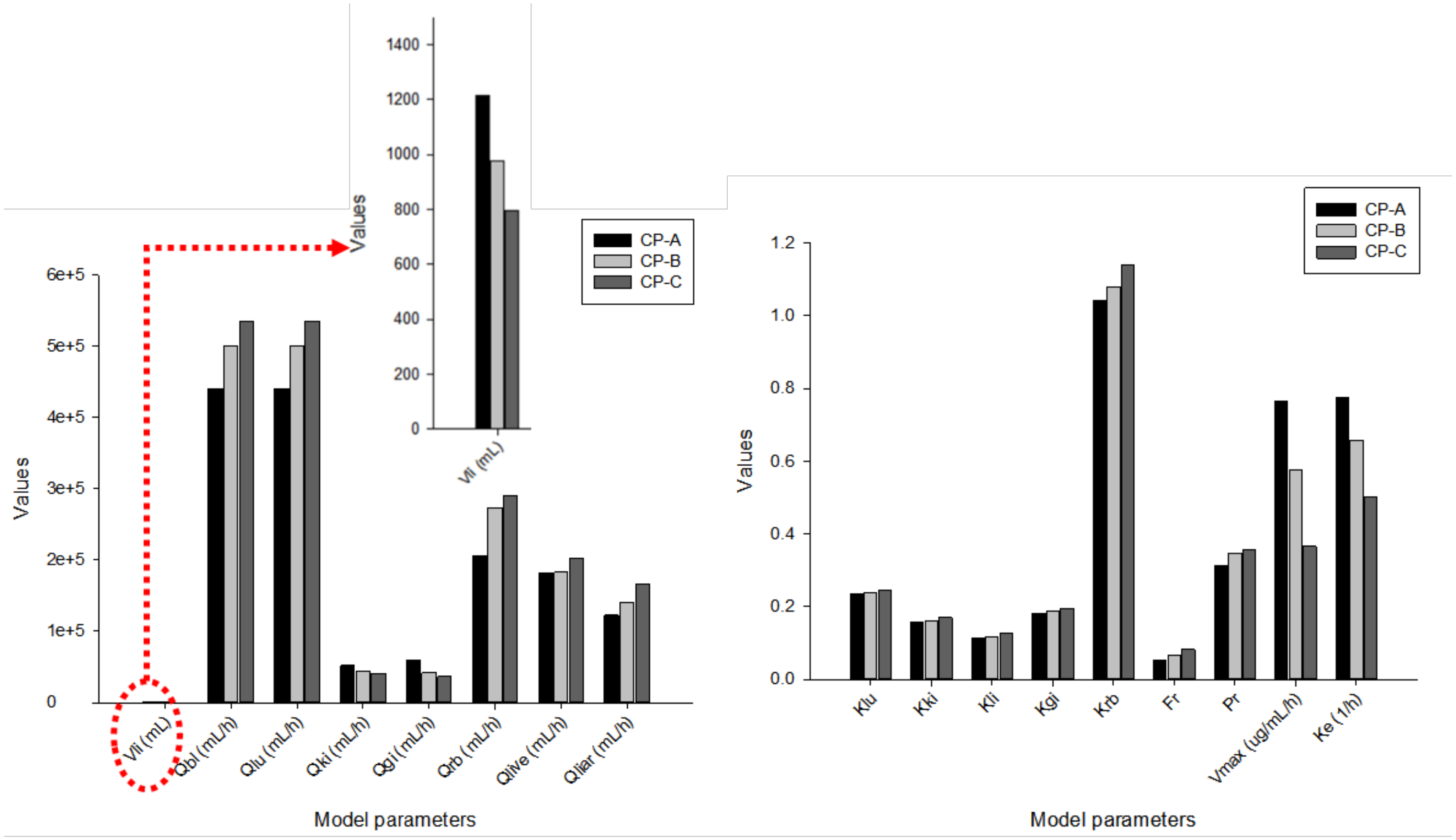

Values
Model parameters
Vli (mL)
Qbl (mL/h)
Qlu (mL/h)
Qki (mL/h)
Qgi (mL/h)
Qrb (mL/h)
Qlve (mL/h)
Qlar (mL/h)
CP-A
CP-B
CP-C
Klu
Kki
Kli
Kgi
Krb
Fr
Pr
Vmax (ug/mL/h)
Ke (1/h)

Figure S10

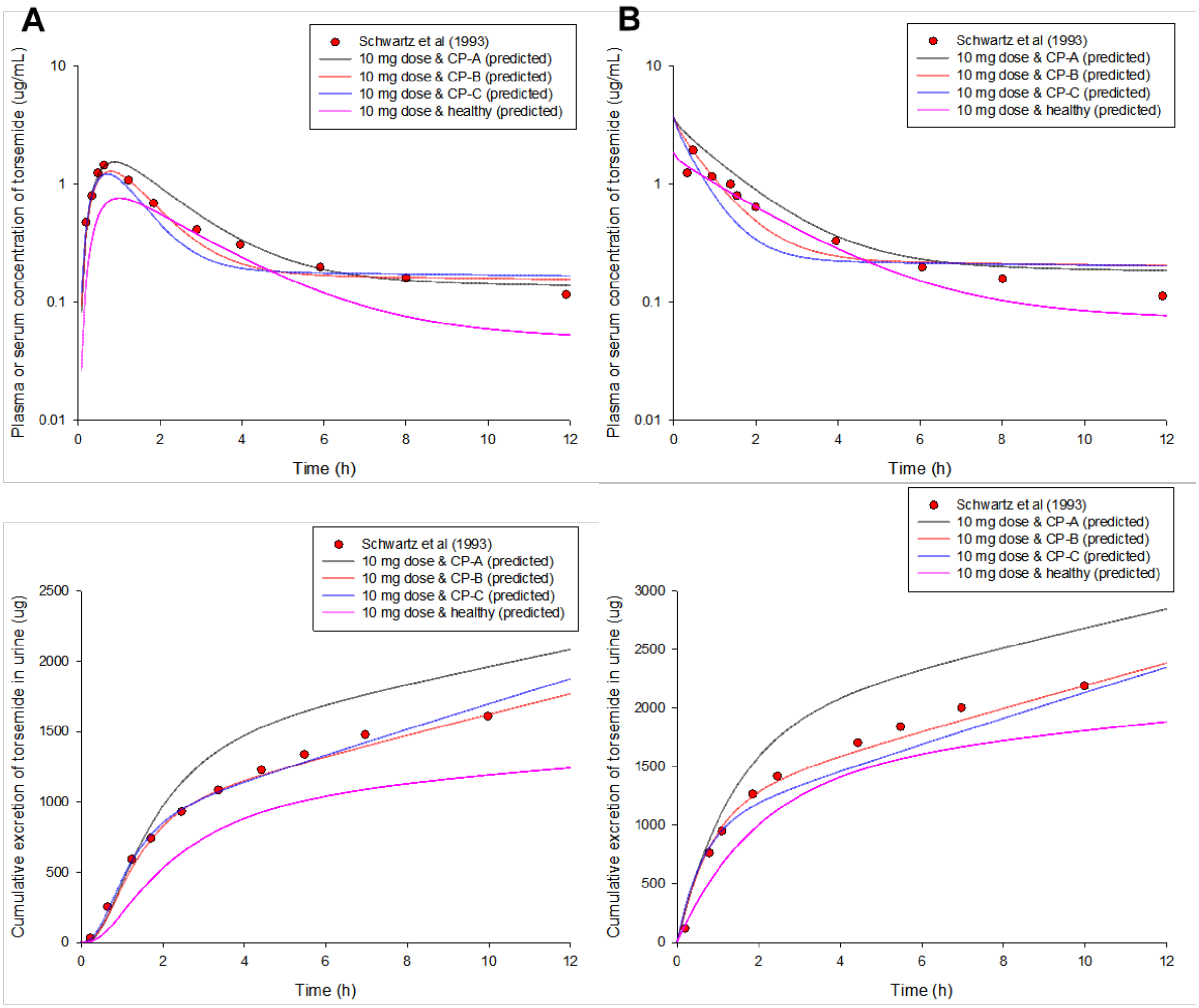
A
B
Schwartz et al (1993)
10 mg dose & CP-A (predicted)
10 mg dose & CP-B (predicted)
10 mg dose & CP-C (predicted)
10 mg dose & healthy (predicted)
Plasma or serum concentration of torsemide (ug/mL)
Cumulative excretion of torsemide in urine (ug)
Time (h)

Figure S11

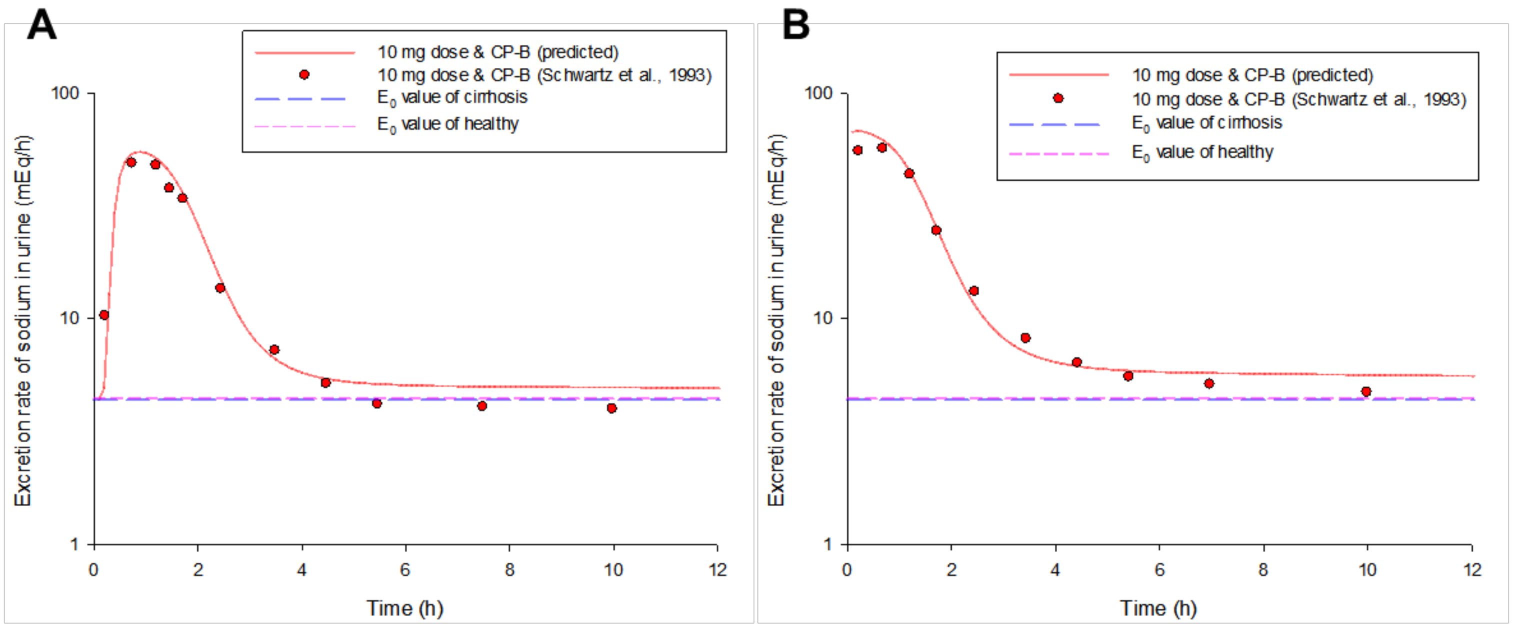
A
B
10 mg dose & CP-B (predicted)
10 mg dose & CP-B (Schwartz et al., 1993)
E_0 value of cirrhosis
E_0 value of healthy
Excretion rate of sodium in urine (mEq/h)
Time (h)

Figure S12

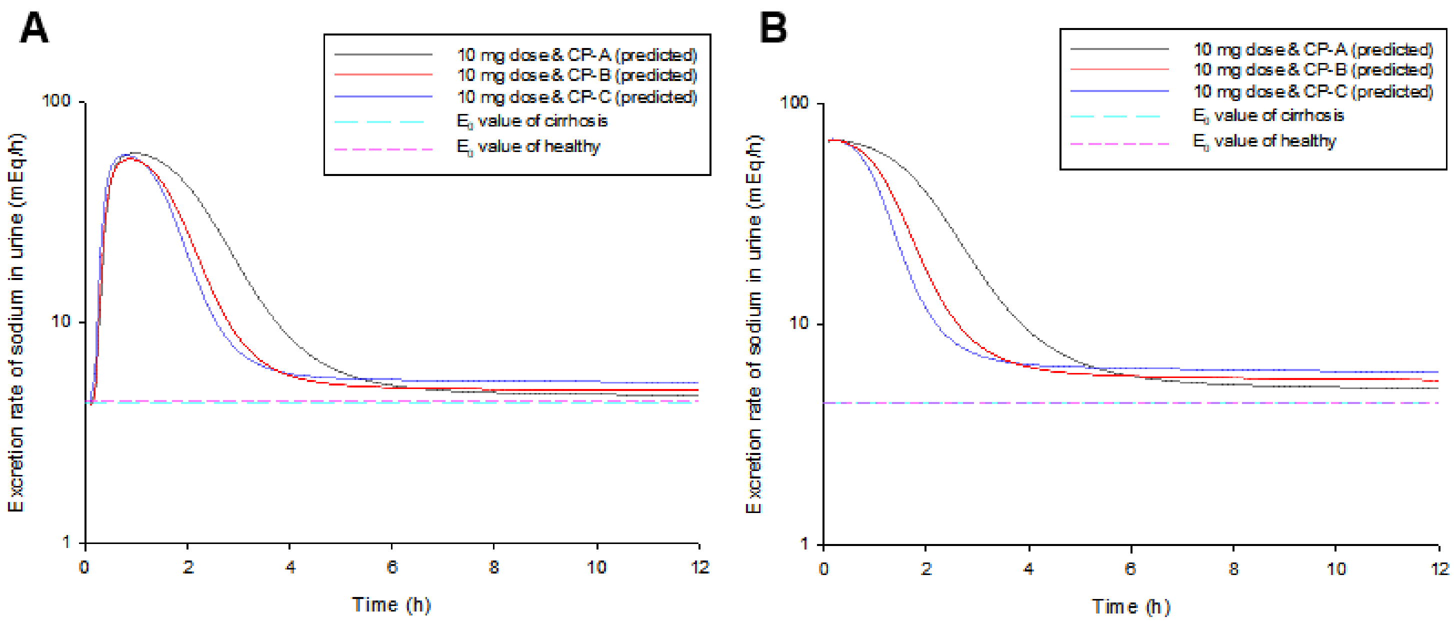
A
10 mg dose & CP-A (predicted)
10 mg dose & CP-B (predicted)
10 mg dose & CP-C (predicted)
E0 value of cirrhosis
E0 value of healthy
Excretion rate of sodium in urine (mEq/h)
Time (h)
B
10 mg dose & CP-A (predicted)
10 mg dose & CP-B (predicted)
10 mg dose & CP-C (predicted)
E0 value of cirrhosis
E0 value of healthy
Excretion rate of sodium in urine (mEq/h)
Time (h)

Figure S13

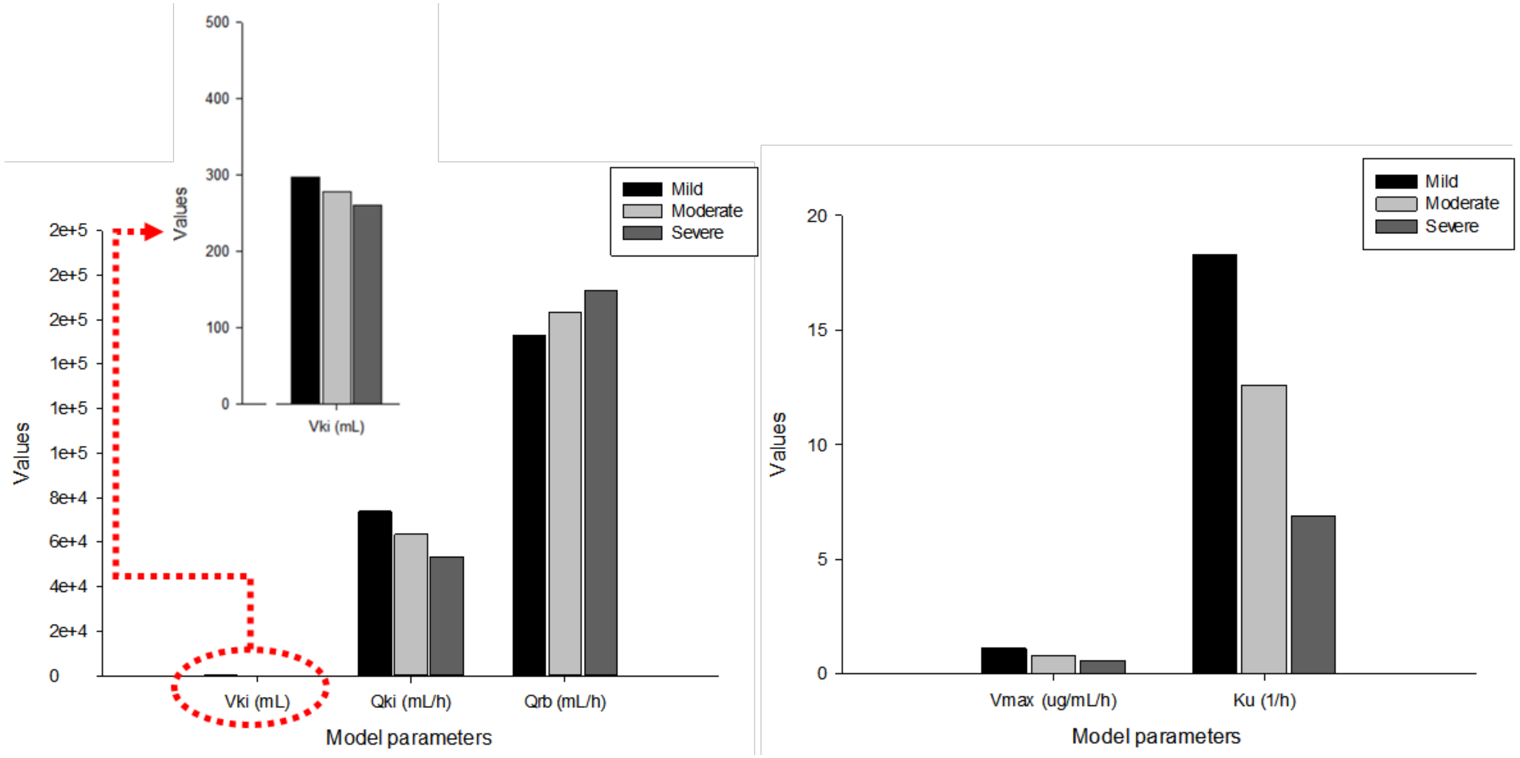

Values
2e+5
2e+5
2e+5
1e+5
1e+5
1e+5
8e+4
6e+4
4e+4
2e+4
0
Values
500
400
300
200
100
0
Vki (mL)
Mild
Moderate
Severe
Vki (mL)
Qki (mL/h)
Qrb (mL/h)
Model parameters
Values
20
15
10
5
0
Mild
Moderate
Severe
Vmax (ug/mL/h)
Ku (1/h)
Model parameters

Figure S14

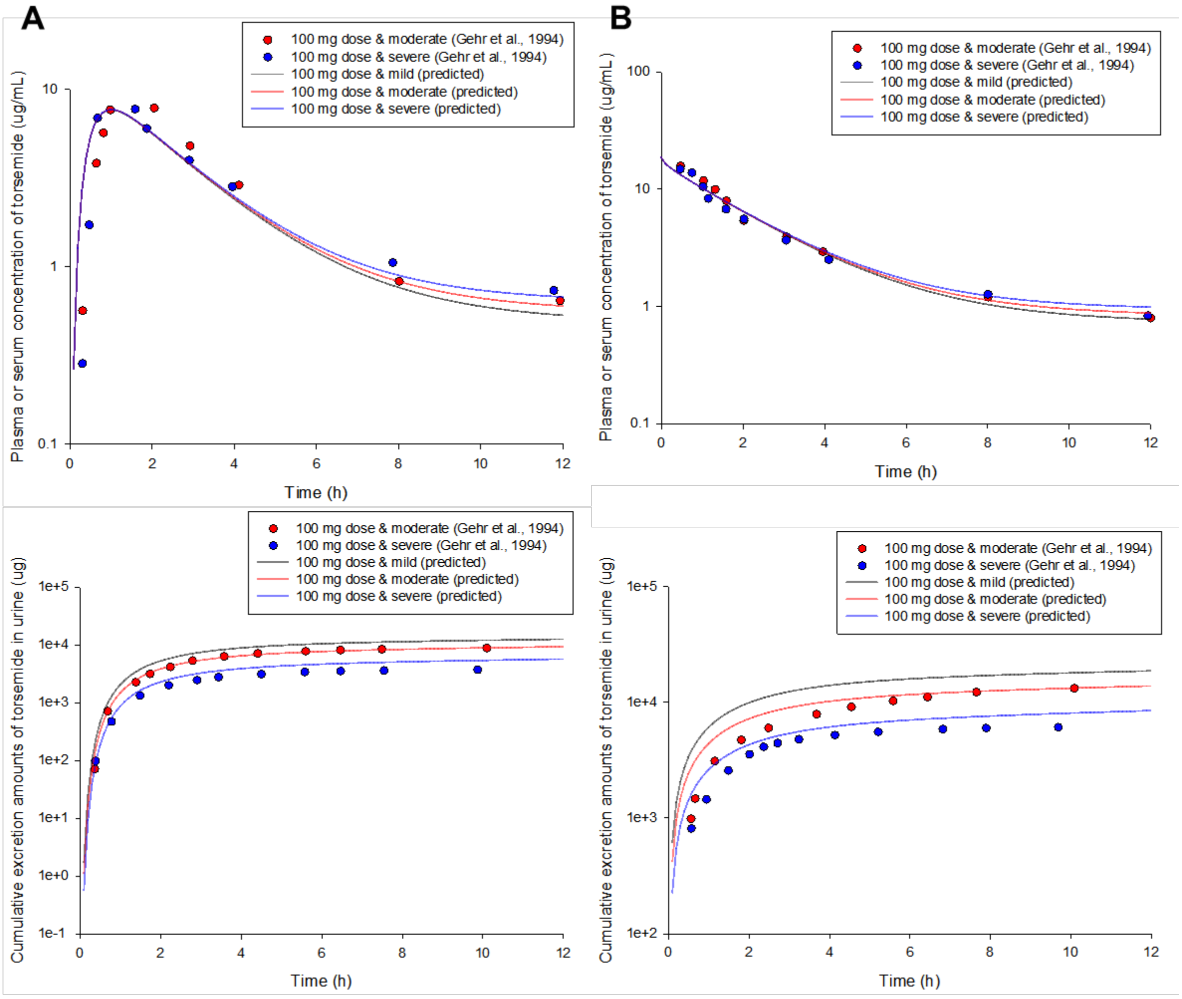

A
B
100 mg dose & moderate (Gehr et al., 1994)
100 mg dose & severe (Gehr et al., 1994)
100 mg dose & mild (predicted)
100 mg dose & moderate (predicted)
100 mg dose & severe (predicted)
Plasma or serum concentration of torsemide (ug/mL)
Cumulative excretion amounts of torsemide in urine (ug)
Time (h)

Figure S15

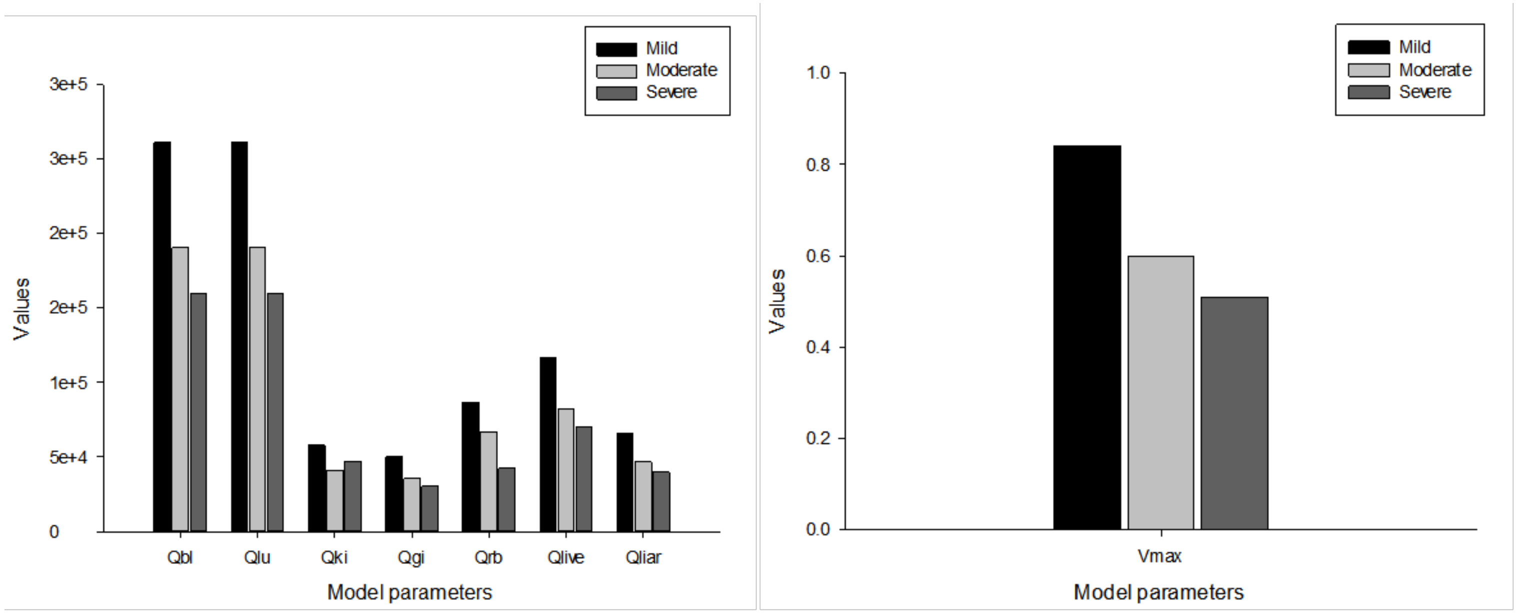

Mild
Moderate
Severe
3e+5
3e+5
2e+5
2e+5
1e+5
5e+4
0
Values
Qbl
Qlu
Qki
Qgi
Qrb
Qlive
Qliar
Model parameters
Mild
Moderate
Severe
1.0
0.8
0.6
0.4
0.2
0.0
Values
Vmax
Model parameters

Figure S16

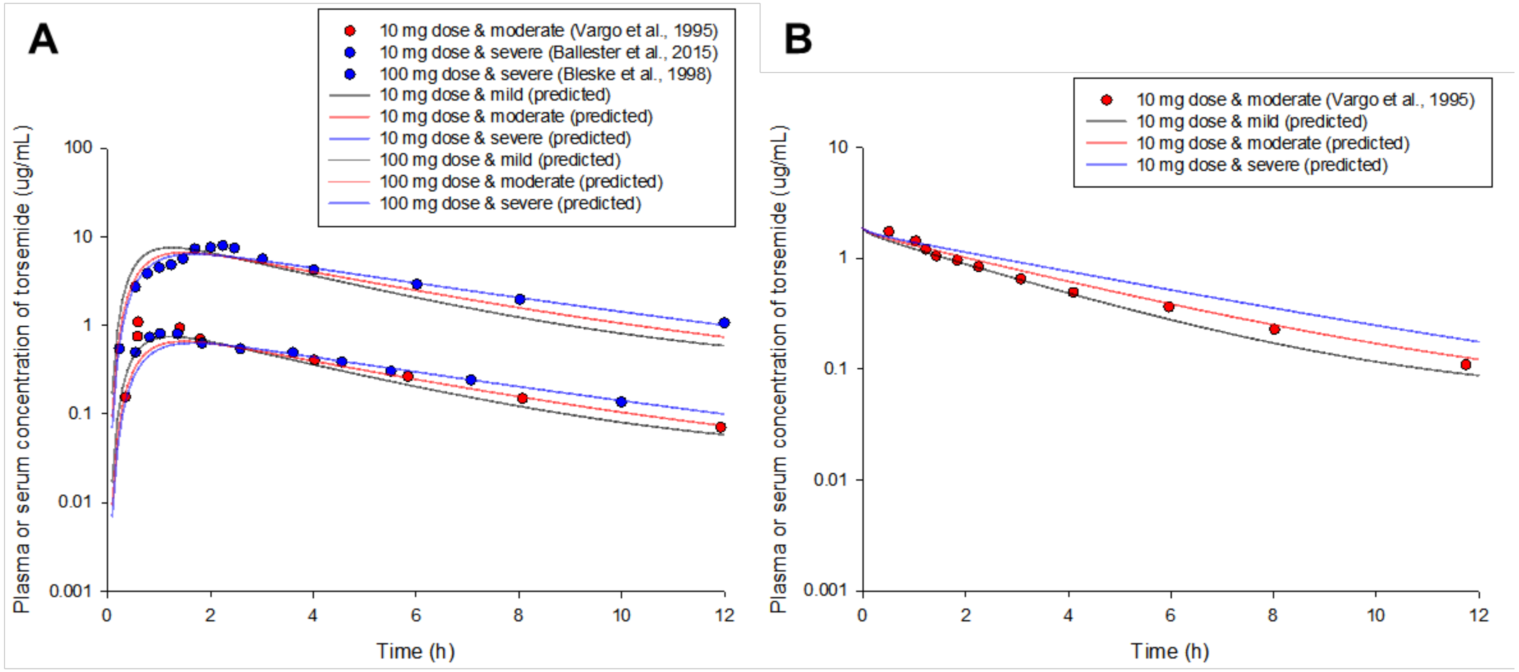
A
10 mg dose & moderate (Vargo et al., 1995)
10 mg dose & severe (Ballester et al., 2015)
100 mg dose & severe (Bleske et al., 1998)
10 mg dose & mild (predicted)
10 mg dose & moderate (predicted)
10 mg dose & severe (predicted)
100 mg dose & mild (predicted)
100 mg dose & moderate (predicted)
100 mg dose & severe (predicted)
Plasma or serum concentration of torsemide (ug/mL)
Time (h)
B
10 mg dose & moderate (Vargo et al., 1995)
10 mg dose & mild (predicted)
10 mg dose & moderate (predicted)
10 mg dose & severe (predicted)
Plasma or serum concentration of torsemide (ug/mL)
Time (h)

Figure S17

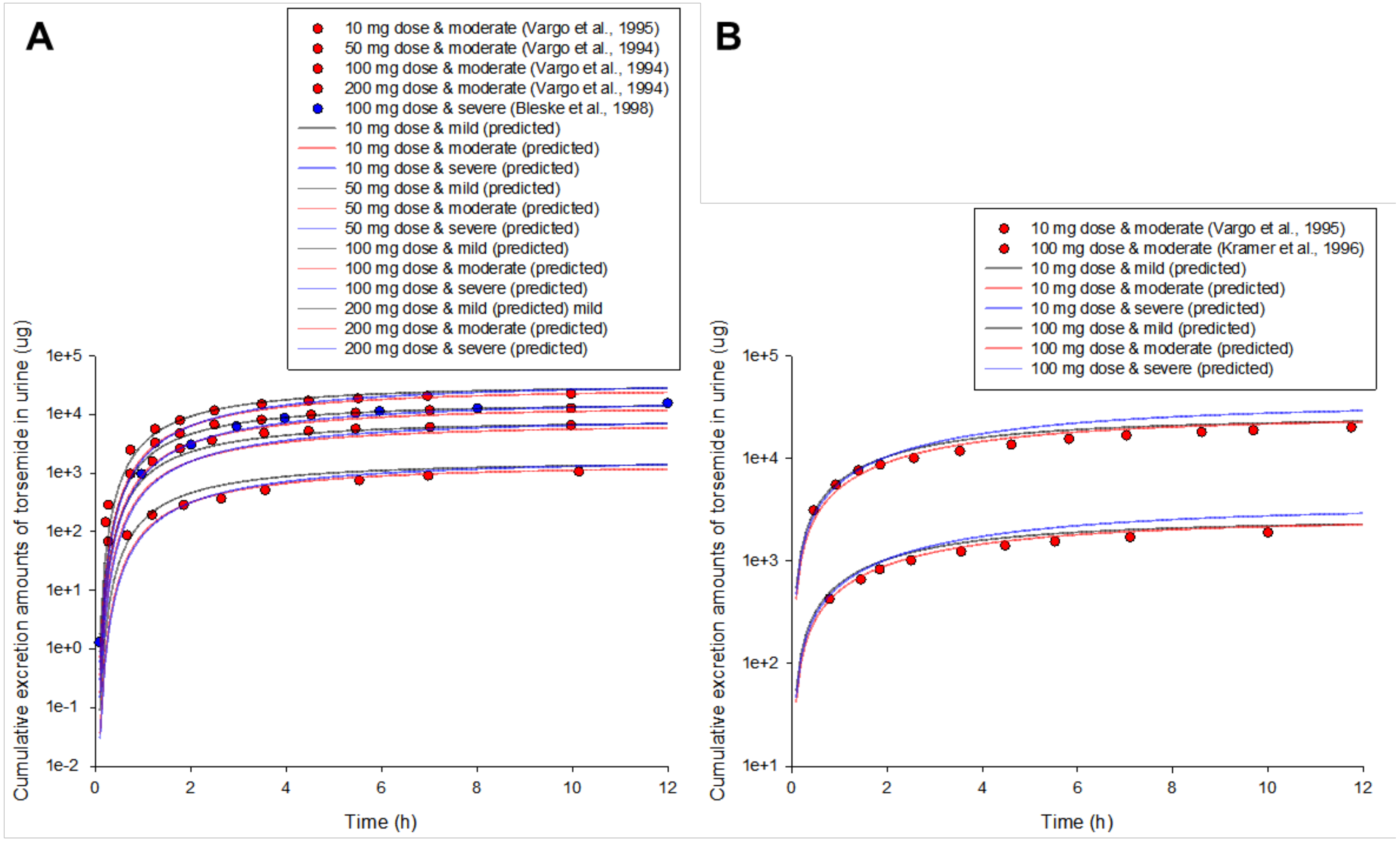
A
10 mg dose & moderate (Vargo et al., 1995)
50 mg dose & moderate (Vargo et al., 1994)
100 mg dose & moderate (Vargo et al., 1994)
200 mg dose & moderate (Vargo et al., 1994)
100 mg dose & severe (Bleske et al., 1998)
10 mg dose & mild (predicted)
10 mg dose & moderate (predicted)
10 mg dose & severe (predicted)
50 mg dose & mild (predicted)
50 mg dose & moderate (predicted)
50 mg dose & severe (predicted)
100 mg dose & mild (predicted)
100 mg dose & moderate (predicted)
100 mg dose & severe (predicted)
200 mg dose & mild (predicted) mild
200 mg dose & moderate (predicted)
200 mg dose & severe (predicted)
Cumulative excretion amounts of torsemide in urine (ug)
Time (h)
B
10 mg dose & moderate (Vargo et al., 1995)
100 mg dose & moderate (Kramer et al., 1996)
10 mg dose & mild (predicted)
10 mg dose & moderate (predicted)
10 mg dose & severe (predicted)
100 mg dose & mild (predicted)
100 mg dose & moderate (predicted)
100 mg dose & severe (predicted)
Cumulative excretion amounts of torsemide in urine (ug)
Time (h)

Figure S18

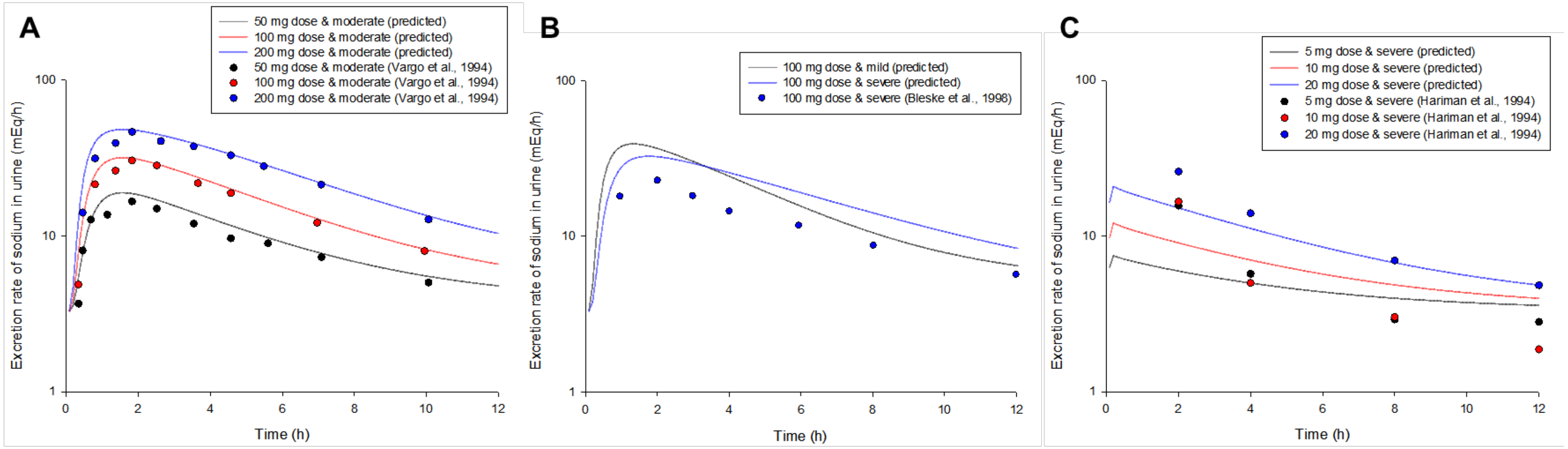
A
50 mg dose & moderate (predicted)
100 mg dose & moderate (predicted)
200 mg dose & moderate (predicted)
50 mg dose & moderate (Vargo et al., 1994)
100 mg dose & moderate (Vargo et al., 1994)
200 mg dose & moderate (Vargo et al., 1994)
Excretion rate of sodium in urine (mEq/h)
Time (h)
B
100 mg dose & mild (predicted)
100 mg dose & severe (predicted)
100 mg dose & severe (Bleske et al., 1998)
Excretion rate of sodium in urine (mEq/h)
Time (h)
C
5 mg dose & severe (predicted)
10 mg dose & severe (predicted)
20 mg dose & severe (predicted)
5 mg dose & severe (Hariman et al., 1994)
10 mg dose & severe (Hariman et al., 1994)
20 mg dose & severe (Hariman et al., 1994)
Excretion rate of sodium in urine (mEq/h)
Time (h)

Figure S19

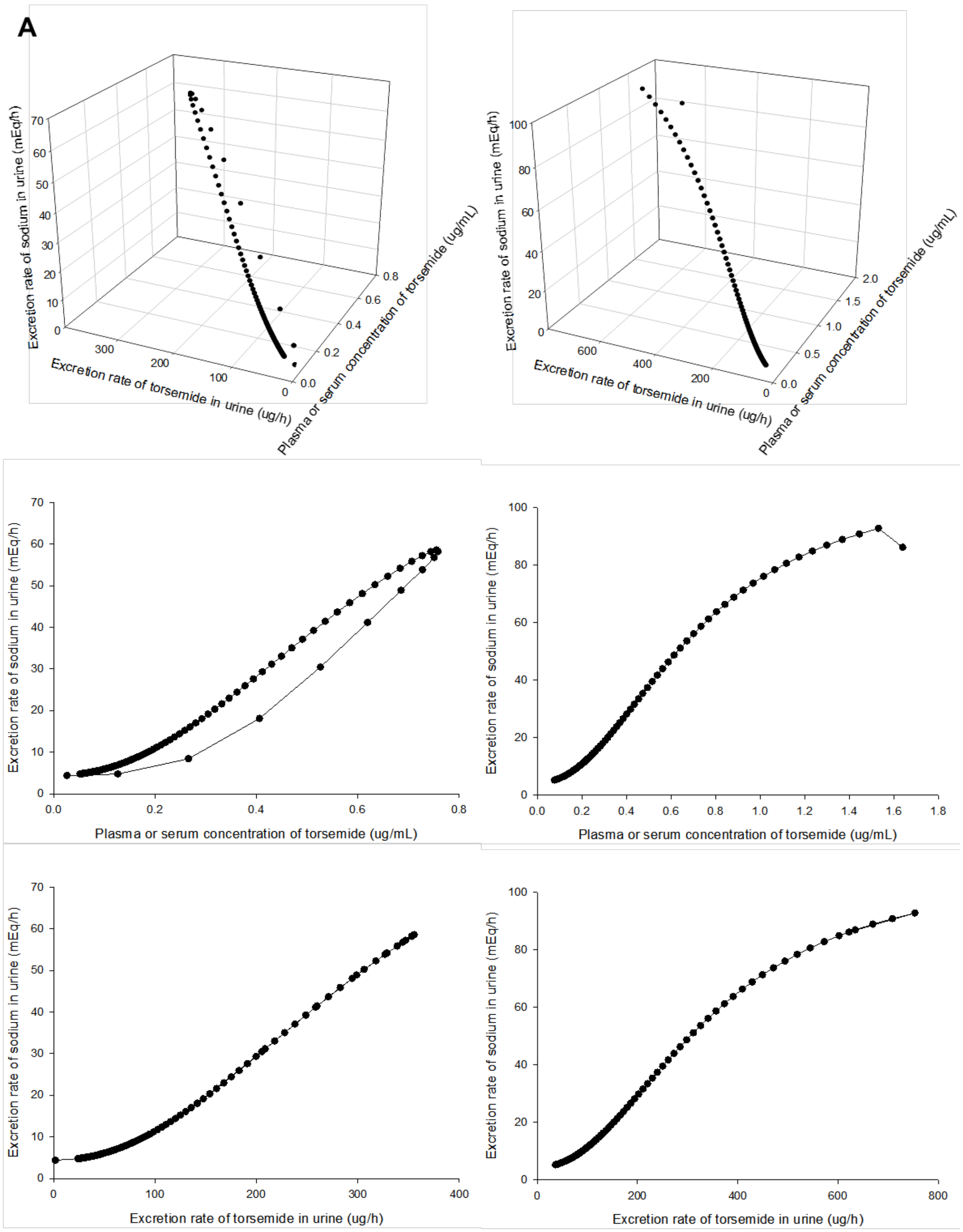

Figure S20 (continued)

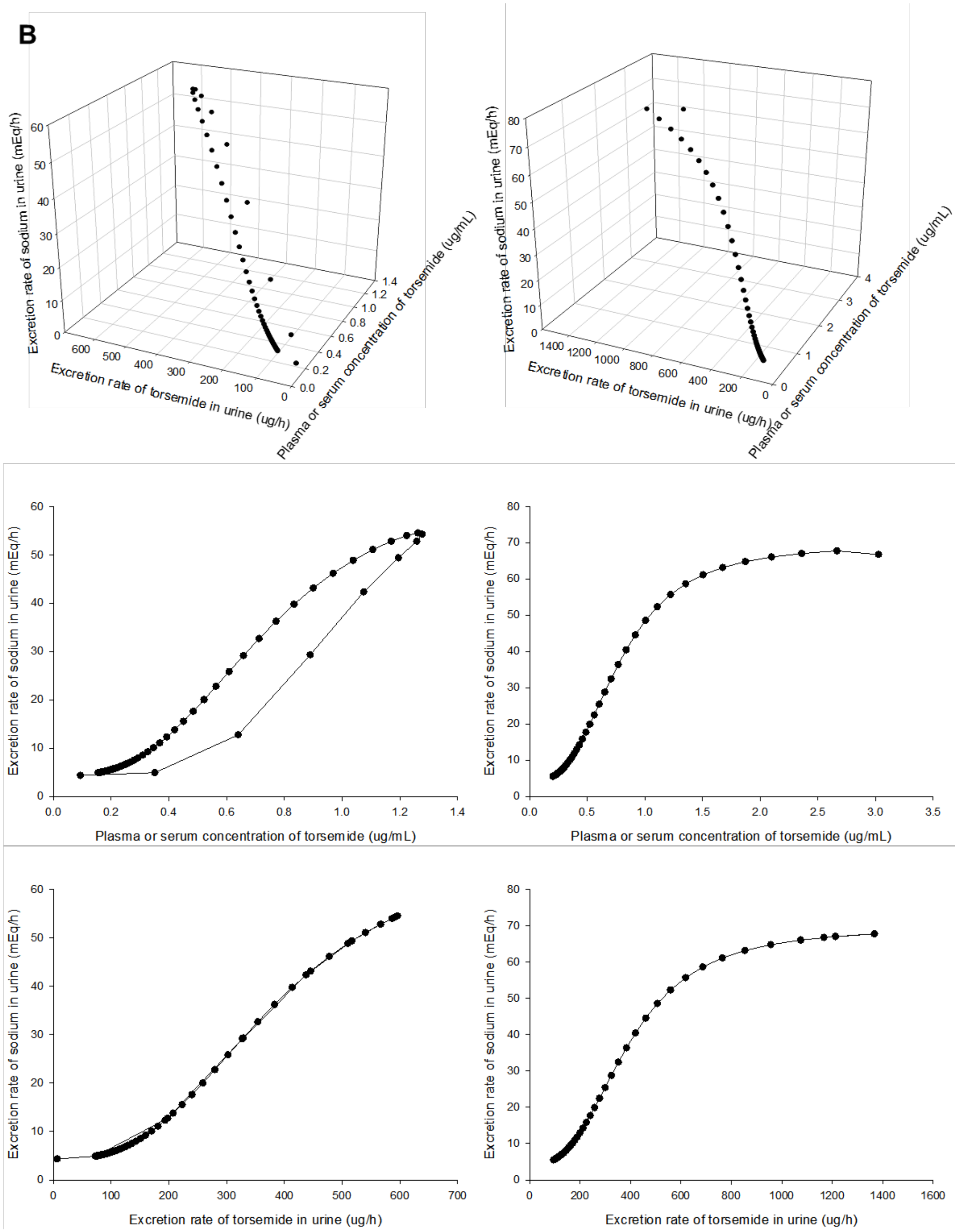

Figure S20 (continued)

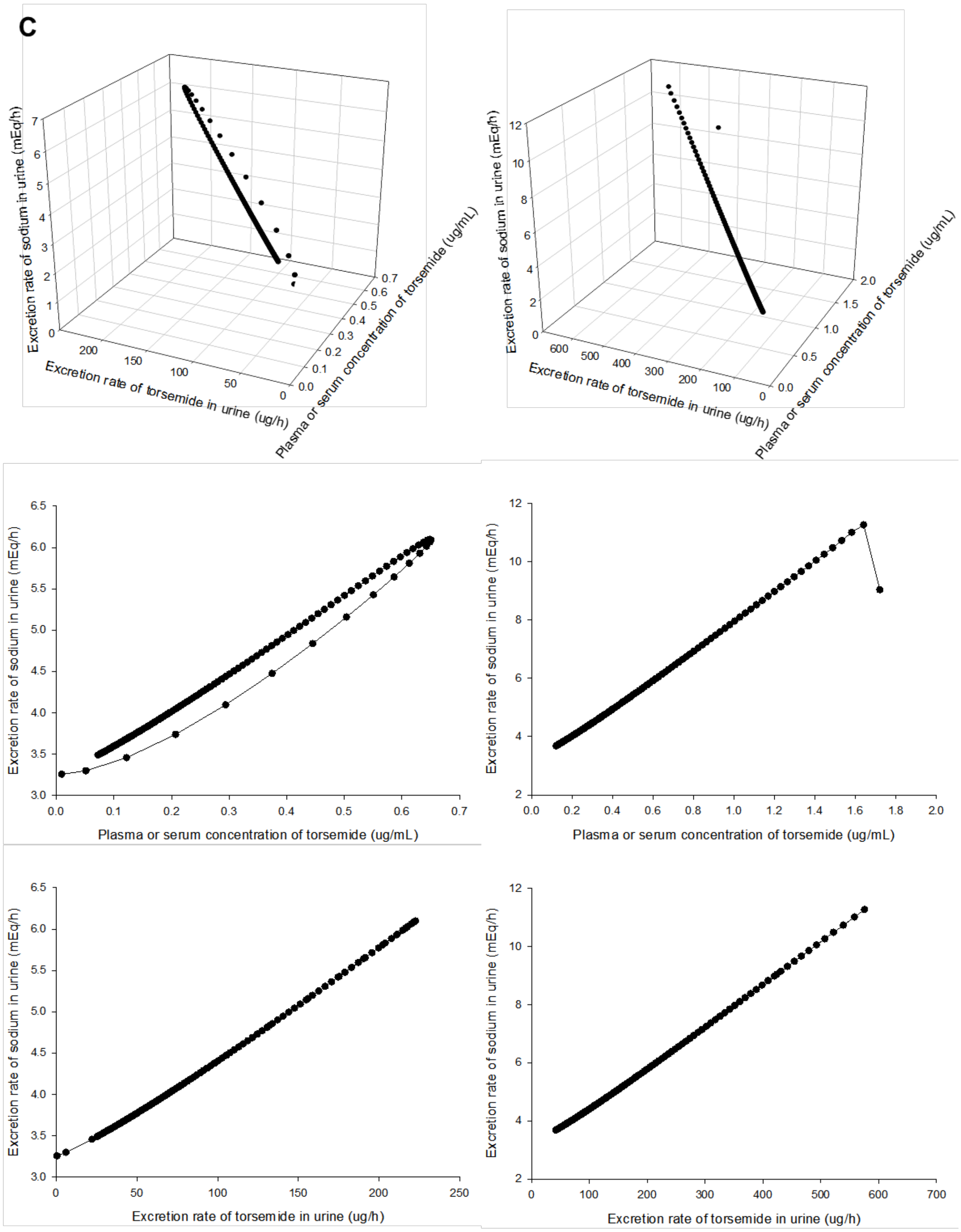
C
Excretion rate of sodium in urine (mEq/h)
Excretion rate of torsemide in urine (ug/h)
Plasma or serum concentration of torsemide (ug/mL)

Figure S20

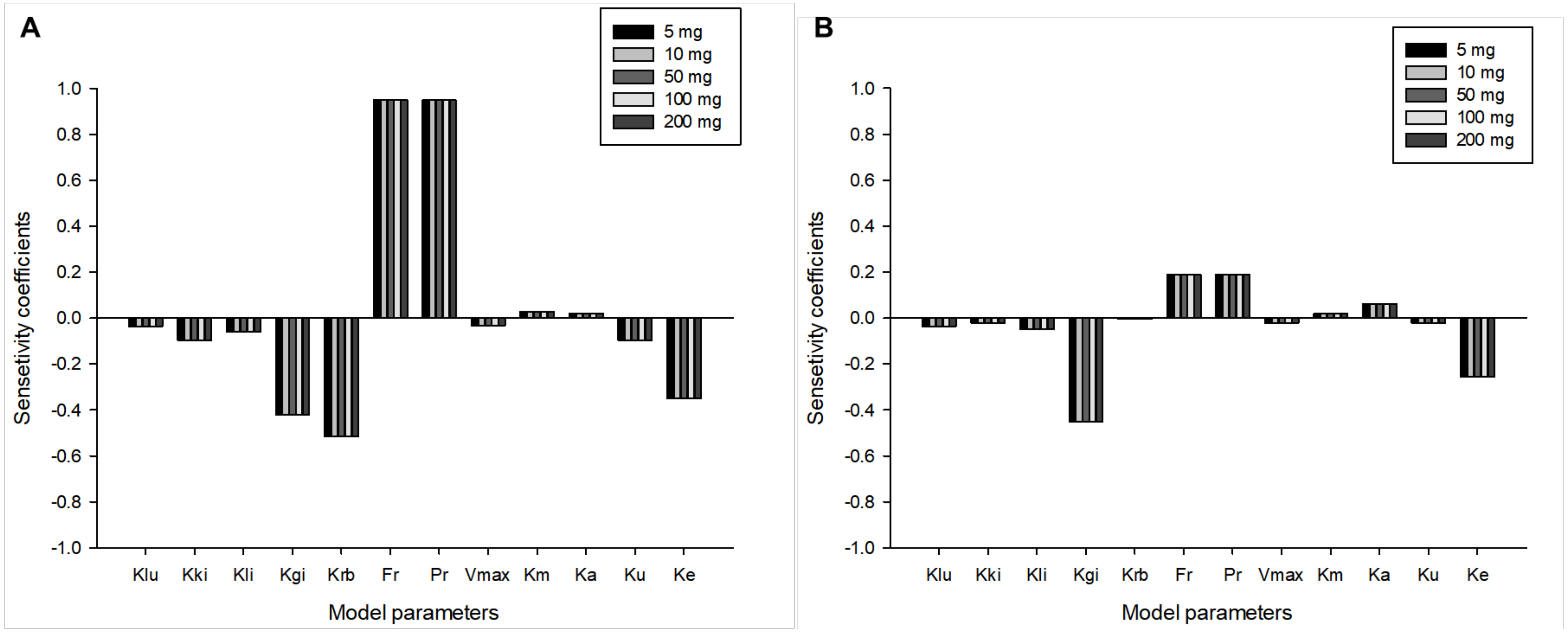
A
5 mg
10 mg
50 mg
100 mg
200 mg
Sensetivity coefficients
1.0
0.8
0.6
0.4
0.2
0.0
-0.2
-0.4
-0.6
-0.8
-1.0
Klu
Kki
Kli
Kgi
Krb
Fr
Pr
Vmax
Km
Ka
Ku
Ke
Model parameters
B
5 mg
10 mg
50 mg
100 mg
200 mg
Sensetivity coefficients
1.0
0.8
0.6
0.4
0.2
0.0
-0.2
-0.4
-0.6
-0.8
-1.0
Klu
Kki
Kli
Kgi
Krb
Fr
Pr
Vmax
Km
Ka
Ku
Ke
Model parameters

Figure S21
